# Brain structural and functional correlates of the heterogenous progression of mixed transcortical aphasia

**DOI:** 10.1007/s00429-023-02655-6

**Published:** 2023-05-31

**Authors:** Diana López-Barroso, José Paredes-Pacheco, María José Torres-Prioris, Guadalupe Dávila, Marcelo L. Berthier

**Affiliations:** 1 Cognitive Neurology and Aphasia Unit, Centro de Investigaciones Médico-Sanitarias (CIMES), University of Malaga, Malaga, Spain; 2 Research Laboratory on the Neuroscience of Language, Faculty of Psychology and Speech Therapy, University of Malaga, Malaga, Spain; 3 Instituto de Investigación Biomédica de Málaga – IBIMA, Malaga, Spain; 4 Department of Psychobiology and Methodology of Behavioural Sciences, Faculty of Psychology, University of Malaga, Malaga, Spain; 5 Radiology and Psychiatry Department, Faculty of Medicine, Universidade de Santiago de Compostela, Santiago de Compostela, Spain; 6 Molecular Imaging Unit, Centro de Investigaciones Médico-Sanitarias (CIMES), General Foundation of the University of Malaga, Malaga, Spain

**Keywords:** DTI-tractography, Functional magnetic resonance imaging, Perisylvian language network, Positron emission tomography, Stroke, Transcortical aphasias

## Abstract

Mixed transcortical aphasia (MTCA) is characterized by non-fluent speech and comprehension deficits coexisting with preserved repetition. MTCA may evolve to less severe variants of aphasias or even to full language recovery. Mechanistically, MCTA has traditionally been attributed to a disconnection between the spared left perisylvian language network (PSLN) responsible for preserved verbal repetition, and damaged left extrasylvian networks, which are responsible for language production and comprehension impairments. However, despite significant advances in in vivo neuroimaging, the structural and functional status of the PSLN network in MTCA and its evolution has not been investigated. Thus, the aim of the present study is to examine the status of the PSLN, both in terms of its functional activity and structural integrity, in four cases who developed acute post-stroke MTCA and progressed to different types of aphasia. For it, we conducted a neuroimaging-behavioral study performed in the chronic stage of four patients. The behavioral profile of MTCA persisted in one patient, whereas the other three patients progressed to less severe types of aphasias. Neuroimaging findings suggest that preserved verbal repetition in MTCA does not always depend on the optimal status of the PSLN and its dorsal connections. Instead, the right hemisphere or the left ventral pathway may also play a role in supporting verbal repetition. The variability in the clinical evolution of MTCA may be explained by the varying degree of PSLN alteration and individual premorbid neuroanatomical language substrates. This study offers a fresh perspective of MTCA through the lens of modern neuroscience and unveils novel insights into the neural underpinnings of repetition.

**Supplementary Information:**

The online version contains supplementary material available at 10.1007/s00429-023-02655-6.

## Introduction

On describing transcortical aphasias, Lichtheim (1885) predicted the coexistence of non-fluent speech and impaired comprehension with the preservation of verbal repetition. This particular combination of symptoms is known as mixed transcortical aphasia. Goldstein (1917, 1948) defined this condition as an “isolation of speech area” disorder resulting from large extrasylvian lesions encircling and disconnecting the structurally spared perisylvian language network (PSLN)—including Broca´s and Wernicke´s areas—from the rest of the cortex (Goldstein 1948; Geschwind et al. 1968; Benson 1979; Kertesz 1979; Damasio 1992). The clinical presentation and neuroanatomical correlates of post-stroke MTCA are heterogeneous. MTCA has been associated with different lesion locations (i.e., extrasylvian areas, perisylvian areas, subcortical nuclei) and language profiles (e.g., lexical versus non-lexical repetition and production) (Coslett et al. 1987; Trojano et al. 1988; Grossi et al. 1991; Berthier et al. 1991). However, MTCA has not been examined under the watchful eye of modern structural and functional neuroimaging techniques, which would increase the existing knowledge on the anatomical basis of these language functions, shed light on the understanding of the heterogeneity of this disorder, and inform the mechanisms of aphasia recovery and treatment (Torres-Prioris et al. 2019b; Stefaniak et al. 2022).

The behavioral profile of MTCA^1^ is characterized by reduced verbal output and poor auditory comprehension and naming in the presence of preserved repetition. Verbal repetition in MTCA is frequently disinhibited and manifested by echolalia and the automatic completion of open-ended sentences (Geschwind et al. 1968; Brown 1975; Assal et al. 1983). Occasionally, picture naming is preserved while performance on word–picture matching tasks can be impaired (Heilman et al. 1976). MTCA is a rare aphasia profile regardless of the type of aphasia test used (e.g., Western Aphasia Battery [WAB], Boston Diagnostic Aphasia Examination [BDAE], clinical impression). As a matter of fact, previous studies with large samples of individuals with aphasia have rarely diagnosed MTCA, with only few cases reported: 4 out of 1200 (0.33%) acute stroke patients studied in Lausanne (Bogousslavsky et al. 1988); 2 out of 444 patients (0.9%) in Benson´s study (Benson 1979); and only 4 out of 208 cases (1.9%) of aphasic Bengali speakers with acute stroke (Lahiri et al. 2020). In addition, no cases of MTCA were documented in a study of 248 Spanish-speaking aphasic patients from Chile (Gonzalez et al. 2021), 226 English-speaking aphasic patients studied in Philadelphia (Landrigan et al. 2021), or in 254 patients from the AphasiaBank database (Clough and Gordon 2020). Therefore, by virtue of its exceptional occurrence, MTCA can be deemed an “orphan” disorder, hindering the acquisition of knowledge on its neural basis, progression (Cauquil-Michon et al. 2011; Flamand-Roze et al. 2011a, b), and response to treatment (Pulvermüller and Schönle 1993; Galling et al. 2014; Torres-Prioris et al. 2019a).

One of the most striking features of this aphasia profile is the relative preservation of verbal repetition abilities. Repetition requires the dynamic interaction between perisylvian frontal and temporal areas, which can be mediated by different white matter tracts. Although the arcuate fasciculus (AF), the main dorsal pathway, has been proposed as the principal route for repetition (Saur et al. 2008), different processes can be used to repeat pseudowords (i.e., without associated meaning) and known words. While the AF is essential for the auditory–motor conversion necessary to repeat a newly heard word (i.e., pseudoword), the ventral pathway, specially the extreme capsule fascicle (ECF), can be utilized to repeat words or phrases with semantic content (Wernicke 1874; Weiller et al. 2021). The ventral pathways comprise the ECF, connecting frontal cortical areas (including Broca´s area) with superior and middle temporal cortices (Weiller et al. 2021) as well as other ventral tracts such as the uncinate fasciculus (UF), and the inferior fronto-occipital fasciculus (IFOF). Although some overlap exists in the frontal cortical regions connecting the ECF and the AF, the AF reaches more posterior and dorsal regions of the frontal cortex (for instance, the premotor cortex), which may explain its primary involvement in pseudoword repetition. The most plausible explanation is that phonological and semantic processes, and the white matter bundles mentioned above, are flexible and dynamically involved during the wide variety of linguistic functions required for proper language functioning (Weiller et al. 2011; López-Barroso et al. 2011). After brain damage, premorbid anatomy and network dynamics will likely influence the resulting acute aphasia profile, while treatment-induced plasticity after brain damage will influence long-term recovery. Previous evidence supports this idea; for instance, preservation of the parieto-temporal posterior AF segment is related to better repetition abilities in patients with primary progressive aphasia (Forkel et al. 2020), while a greater volume of the right long AF segment may be advantageous in case of left stroke resulting in aphasia, supporting language recovery (Forkel et al. 2014). Finally, preserved repetition in MCTA has been related to a voluminous right AF (Berthier et al. 2012). Therefore, it is likely that the pathways that support verbal repetition in MCTA are variable, as is the case in other linguistic functions in the healthy brain (López-Barroso et al. 2011; López-Barroso and de Diego-Balaguer 2017).

The classical brain-language account of MTCA attributed to isolation of the PSLN is exemplified by a person who suffers two or more simultaneous or successive left hemisphere lesions; one anterior involving the dorsolateral or mesial frontal cortices, which accounts for non-fluent language production, and another involving the posterior temporal cortex and/or inferior parietal areas, which may account for lexical-semantic deficits (Geschwind et al. 1968; Assal et al. 1983; Bogousslavsky et al. 1988; Ibayashi and Soma 1997; Maeshima et al. 2002; Cauquil-Michon et al. 2011; Flamand-Roze et al. 2011a, b; Rosca and Simu 2015). However, MTCA has also been reported after a single stroke involving the frontal cortex (Rapcsak et al. 1990), the left thalamus (Nitrini et al. 2019), large parts of the perisylvian cortex (Pulvermuller et al. 1993), or the medial parietal cortex (Ross 1980). The claim that the functional mechanism of isolation of the PSLA explains MTCA has been accepted as a putative mechanism to explain MTCA (Geschwind et al. 1968; Heilman et al. 1976; Kertesz 1979; Assal et al. 1983; Damasio 1992), yet the evidence confirming its validity is scant and elusive. For example, studies on MTCA have shown inconsistencies in the degree of sparing of the PSLN (Bogousslavsky et al. 1988; case 2 in Brown 1975; Maeshima et al. 2002) or in the resulting language profile (Berthier et al. 1991; Pulvermüller and Schönle 1993). Thus, alternative explanations to the isolation mechanism have been proposed to account for the preservation of verbal repetition in cases of affectation of the PSLN and the AF, which emphasize the role of the right hemisphere (Niessl von Mayendorf 1911; Stengel 1947; Grossi et al. 1991; Berthier et al. 1991; Pulvermuller et al. 1993), both hemispheres (Brown 1975; Ohyama et al. 1996; Berthier 1999), or the remnants of the left PSLN connected through the ventral pathway (Weiller et al. 2011, 2021). In summary, these controversial data question the validity of the isolation of the PSLN as the sole mechanism underlying MTCA and raise inquiries on whether the complete integrity of the cerebral cortex and white matter tracts composing the PSLN is necessary to explain the syndrome.

Additionally, the importance of other cognitive domains in MCTA must be considered. In fact, Goldstein (1948) hypothesized that transcortical sensory aphasia resulted from damage “to the relation between the instrumentalities [of language] and the non-language mental performances”. This early hypothesis can be accommodated with the suggested altered interaction between a multi-demand (MD) cognitive system and the left-dominant language-specific PSLN in MTCA (Hertrich et al. 2020). The MD system comprises different networks in both hemispheres related to various cognitive operations, including attention, working memory, inhibitory control of automatic responses, and selection serving multiple cognitive functions (Duncan 2010; Brownsett et al. 2014; Geranmayeh et al. 2017). These MD networks include extrasylvian fronto-parietal areas (executive network) related to superordinate control of language and communication, and a cingulo-opercular (salience) network, processing salient information. The PSLN occupies the left core fronto-temporal cortex surrounding the Sylvian fissure, in the vicinity of MD networks or intermingled with them (Fedorenko et al. 2012; Campbell and Tyler 2018; Braga et al. 2020; Diachek et al. 2020; Fedorenko and Blank 2020). In normal conditions, both networks dynamically cooperate during complex language tasks that require executive control. Brain damage causing MCTA may involve the bordering areas of the PSLN and the extrasylvian regions that support MD functions, thereby altering the dynamic interplay between MD cognitive networks (Duncan 2010) and spared areas of the PSLN (Fedorenko et al. 2012; Diachek et al. 2020; Fedorenko and Blank 2020). This alteration can result in impaired executive control over the PSLN, and the unmonitored activity of the still functional PSLN can be clinically manifested by disinhibition of inappropriate behavioral responses such as uncontrolled repetition, echolalia, and automatic completion of open-ended sentences and clichés in MTCA (Stengel 1947; Geschwind et al. 1968; Berthier et al. 2017). Therefore, studying MTCA associated with extrasylvian lesions accompanied by variable PSLN affectation can provide valuable data on the respective participation of these segregated networks in different types of language deficits. For example, since MTCA is frequently associated with disinhibition of repetition skills (i.e., echolalia), its study can inform how the impairment of control-related systems exerts a permissive role in disinhibiting automatic verbal responses (Berthier et al. 2017; Darda and Ramsey 2019).

To the best of our knowledge, no modern neuroimaging techniques have been used to investigate the neural underpinnings of MTCA. In this context, we have the opportunity to examine the functional and structural status of the PSLN in four cases with initial presentation of MTCA, as well as to study its heterogeneous clinical progression. The present study employs neuroimaging methods to investigate the neural basis of MTCA including: (1) task-related functional magnetic resonance imaging (fMRI) to explore brain regions functionally involved in verbal repetition; (2) resting-state fMRI to identify the language and MD resting-state networks; (3) diffusion tensor imaging (DTI-tractography) to assess structural disconnection of the dorsal and ventral language streams; and (4) ^18^Fluorodeoxyglucose positron emission tomography (^18^FDG-PET) to examine the metabolic state of the PSLN during rest. Measuring resting metabolic activity in areas of the PSLN and neighboring regions showing no structural damage seems appropriate because some cases of MTCA have also been described combining structural damage of premotor and inferior frontal cortical areas (Rapcsak et al. 1990; Maeshima et al. 1996) with metabolic derangements in the parietal cortex, most likely due to connectional diaschisis (a form of functional diaschisis distant from the structural damage that occurs independently of contextual stimulation) (Campo et al. 2012; Carrera and Tononi 2014). This form of functional disconnection has been applied to other clinical profiles characterized by cognitive deficits in absence of visible structural damage (Jha et al. 2020).

## Method

### Participants

Participants were four right-handed native Spanish speakers who developed acute MTCA following two or multiple stroke lesions affecting the left frontal and temporo-parietal cortices with different degrees of involvement of the left PSLN. In the present study, post-stroke stages are defined as follows: acute/subacute (weeks 1–12), and chronic (> 12 weeks) (Berthier et al. 2014). All patients were evaluated in the acute/subacute stage while in the hospital or at follow-up visits with bedside methods performed by the attending neurologists and speech therapists, who established the diagnosis of MTCA. Patient 3 (P3) suffered two stroke episodes and was evaluated with bedside methods in both of them, but he met the diagnosis of MTCA after the second stroke lesion. In the early chronic period (4–10 months after stroke), P1 and P4 were inpatients in rehabilitation centers, where they were additionally evaluated with the Boston Diagnostic Aphasia Examination (BDAE) or the BDAE Short Form (Goodglass and Kaplan 1983; García-Albea et al. 1996), confirming their diagnosis of MTCA. Table S1 shows demographic data and information regarding the classification of acute/subacute and chronic aphasia.

The language deficits of P1 and P4 improved with inpatient intensive aphasia therapy (~ 5 h/week), evolving to less severe aphasia profiles in the chronic stage (anomic aphasia in P1 and latent aphasia with narrative impairment in P4). Only P2 met the diagnostic criteria for MTCA in the chronic stage. P3 did not receive aphasia therapy, and his language deficits improved spontaneously, evolving to anomic aphasia with features of dynamic aphasia. At the chronic language evaluation, all patients still showed residual features of transcortical aphasia, including disinhibited repetition manifested by frequent instances of automatic and mitigated echolalia on completing auditory comprehension subtests (Yes–No Questions, Auditory Word Recognition, and Sequential Commands) of the WAB-R (Table S1). The patients were screened for a clinical trial for which they did not meet one of the inclusion criteria (affected repetition) (EudraCT:2017–002,858-36; ClinicalTrials.gov identifier: NCT04134416). The patients and/or their careers signed written informed consent. In addition, 25 healthy adult controls underwent ^18^FDG-PET acquisition (15 males; age mean: 58.25 ± 12.72 years; range: 48–67 years). See Supplementary Method for details on ^18^FDG-PET acquisition parameters.

### Background language, cognitive testing, and neuroimaging in the chronic stage

All patients underwent a behavioral evaluation consisting of language and cognitive testing and a neuroimaging evaluation with structural and functional MRI and ^18^FDG-PET performed at least 6 months after stroke onset (range: 7–36 months) (Table S1). By that time, P2 still had MTCA, but language deficits in the other three patients evolved into less severe aphasic syndromes. All patients still had the usual dissociation between impaired verbal communication, comprehension or both and nearly intact word and sentence repetition described in transcortical aphasias (Albert et al. 1981; Berthier 1999).

Each patient's demographic, clinical, language, and cognitive information is reported in Supplementary Results (Case presentations and Tables S1 and S2). The behavioral and neuroimaging evaluations in the chronic stage were performed in our Research Unit when the four participants were ambulatory patients. The aphasia diagnosis at this stage was established with the Western Aphasia Battery-Revised (WAB-R) (Kertesz 2007) and indicated anomic aphasia with echolalia in P1 (Table S1), severe MTCA in P2 (Table S1), anomic aphasia with features of dynamic aphasia (Robinson et al. 1998) in P3 (Tables S1 and S2), and latent aphasia with elements of a narrative impairment (Alexander 2006) in P4 (Tables S1 and S2). All patients showed moderate to severe impairment in the frequency and quality of everyday verbal communication measured with the Communicative Activity Log (Pulvermuller and Berthier 2008). Subtests of the Spanish version of Psycholinguistic Assessments of Language Processing in Aphasia (PALPA) (Kay et al. 1992) (Evaluación del Procesamiento Lingüístico en la Afasia—EPLA; Valle and Cuetos 1995) were also administered. The Spanish version of the PALPA (i.e., EPLA) is a standardized battery that assesses processing in different language components that may be independently impaired. It comprises 58 subtests that assess language recognition, comprehension, and production abilities. However, EPLA is not designed to be administered in entirety to an individual, but rather the assessment should be tailored and restricted to the appropriated subtests (Valle and Cuetos 1995). Thus, specific subtests aimed to evaluate input phonology (word minimal pairs), lexical access (auditory lexical decision) and comprehension (spoken word–picture matching and sentence comprehension) were administered. Repetition subtests were also used to assess the effect of psycholinguistic variables (e.g., number of phonemes, semantic content, frequency, imageability and frequency) in repetition abilities (see Table S1). EPLA-PALPA showed preserved input phonology and lexical-semantic processing for single words in P1, P3, and P4 but sentence comprehension impairments in P1 and P3. Some EPLA-PALPA subtests were challenging to administer in P2 due to the interference caused by severe echolalia and linguistic anxiety (see Torres-Prioris et al. 2019a). Performance in word repetition was better than in non-word repetition, and performance in experimental tests of sentence repetition (clichés and non-clichés) was preserved in the four patients. Completion of high-constraint and low-constraint sentence frames omitting the final word was impaired in P1 and P2 and preserved in P3 and P4. Auditory short-term memory for digits was mildly reduced in P2, P3, and P4 and working memory was altered in all patients.

Testing of non-verbal reasoning with the Raven Colored Progressive Matrices showed average performance in all patients (Measso et al. 1993). Only P3 and P4 were evaluated with tests tapping attention and executive functions. The Controlled Oral Word Association Task (Borkowski et al. 1967) showed reduced phonemic fluency in both of them, and performance on part B of the Trail-Making Test (Reitan 1958) was impaired in P3, while both parts were normal in P4. Inhibition of automatic verbal responses in P3 and P4, evaluated with the Hayling Sentence Completion Test (Burgess and Shallice 1996; Pérez-Pérez et al. 2016), showed impaired performance. Instances of automatic and mitigated echolalia were documented during the administration of the auditory comprehension subtests of the WAB-R in all patients. Sentence completion was evaluated with the Sentence Completion section of the WAB-R and, in P3 and P4, also with an experimental battery of dynamic aphasia tests (Robinson et al. 1998) (Table S2).

### Neuroimaging

#### Neuroimaging acquisition and preprocessing

P2, P3, and P4 underwent a multimodal 3 T MRI session including structural (T1-weighted image, diffusion tensor imaging [DTI]) and functional (fMRI during covert word and pseudoword repetition; and resting-state fMRI]) sequences. P1 could not undergo the 3 T MRI session due to an incompatible clip used to obliterate a saccular aneurysm, but instead, she underwent a structural 1.5 T MRI. In addition, P1, P2, P3, and P4 and a control sample of 25 healthy adult subjects underwent a ^18^FDG-PET session. Detailed information on neuroimaging acquisition and preprocessing of the data is reported in the Supplementary Method (Neuroimaging acquisition and Neuroimaging preprocessing).

#### Lesion delineation and lesion load

Lesions were manually delineated for each patient by the first author (DL-B) and supervised by the last author, a trained neurologist (MLB). Lesions were drawn on the axial slices directly over the T1-images (for P2, P3 and P4) and the T2-image (for P1) in the native space using MRICRON software (Rorden and Brett 2000). The lesion masks were normalized to the standard MNI space (see Supplementary Methods) and the degree of overlap of each lesion with regions belonging to the perisylvian area was calculated. The perisylvian area was defined by joining the following left hemisphere anatomical areas: Rolandic_Oper, Frontal_Inf_Tri, Frontal_Inf_Oper, Precentral, Postcentral, SupraMarginal, Angular, Temporal_Sup, Temporal_Mid, and Insula from the AAL atlas as provided by the Wake Forest University (WFU) Pickatlas toolbox (https://www.nitrc.org/projects/wfu_pickatlas/), using SPM12. Finally, the volume of the normalized lesion masks was extracted using the fslstats command of FMRIB software library (FSL; https://fsl.fmrib.ox.ac.uk/fsl/fslwiki/FSL).

#### Task-related fMRI analysis

The general linear model was applied to find activations related to the following contrasts: word repetition > rest and pseudoword repetition > rest. The mean time series of the blood-oxygen-level-dependent (BOLD) signal in white matter and cerebrospinal fluid and realignment parameters was included in the model as covariates to remove signal from non-neural sources. A normalized cerebral mask excluding the lesion was used for the estimation of the activation maps. Activations are reported using a statistical threshold of *p* < 0.05, FWE corrected at the cluster level, with a minimum cluster size of 20 voxels. Lateralization indexes (LI) were determined for each patient and contrasted by calculating a weighted LI using a validated threshold-independent method resistant to outliers and stable against noise (AveLI; Matsuo et al. 2012). AveLi ranges from 1 (extreme left lateralization) to − 1 (extreme right lateralization), with values between 0.2 and − 0.2 considered bilateral. Activation maps in standard MNI space are rendered over a mesh using the visualization software Surf Ice (https://www.nitrc.org/projects/surfice/).

#### Resting-state fMRI: functional connectivity analysis

Functional connectivity among a set of regions of interest (ROIs/seeds) and every voxel in the brain was studied by performing a seed-based correlational approach with CONN toolbox (Nieto-Castanon 2020). Functional connectivity analysis of three commonly studied resting-state networks (Smith et al. 2009) was performed using three seeds. The networks’ ROIs were selected from the default CONN network´s cortical atlas (derived initially from independent component analyses of 497 subjects from the HCP dataset) (Whitfield-Gabrieli and Nieto-Castanon 2012) of the language network: left and right inferior frontal gyrus—IFG—(ROI 1:left IFG [center coordinate: − 52, 26, 2] and ROI 2: right IFG [center coordinate: 54, 28, 2]); the salience network: anterior cingulate cortex—AAC—(ROI 3: AAC [center coordinate: 0, 22, 35]); and the executive network: bilateral posterior parietal cortex—PPC—(ROI 4: left PPC [center coordinate: − 46, − 58, 50] and ROI 5: right PPC [center coordinate: 52, − 52, 46]) (see Figure S1). Pearson´s correlation coefficients were calculated between the average time series within each ROI and the time series of all other voxels in the brain. First-level Pearson´s correlations for each ROI are reported for each suprathresholded area using a *p* < 0.05, FDR corrected.

Further, to explore the status of the resting-state networks of the three patients, the resulting connectivity maps were spatially cross-correlated with the maps of healthy participants obtained from Neurosynth (https://neurosynth.org/locations/). Specifically, cross-correlation analyses were performed between each statistically significant seed-based correlation map and the functional connectivity map for each ROI obtained from Neurosynth. Neurosynth allows the introduction of coordinates in MNI space and returns coactivation maps for that seed region based on data from 1.000 participants (Yeo et al. 2011). Cross-correlations were performed with the FSL command “fslcc”. All functional connectivity maps were rendered over a mesh using the visualization software Surf Ice (https://www.nitrc.org/projects/surfice/).

#### ^18^FDG-PET analysis

Statistical analysis was carried out with Statistical Parametric Mapping software (SPM12, http://www.fil.ion.ucl.ac.uk/spm/). Areas of hypometabolism were estimated for each patient with the contrast: patient < healthy controls; and areas of hypermetabolism were calculated using the contrast: patient > healthy controls, using a two-sample *t *test. A statistic threshold of *p* < 0.05, FWE corrected at cluster level was applied. The hypometabolism, the hypermetabolism, the lesion, and the left perisyvian area were mapped onto a template surface using Freesurfer (https://surfer.nmr.mgh.harvard.edu/). Areas included in the perisylvian mask used are described in Sect.  Lesion delineation and lesion load.

#### White matter tractography

Trackfiles derived from spherical deconvolution reconstruction method (see Supplementary Method) were imported to Trackvis software to perform the virtual dissections of the tracts of interest: the three segments (anterior, posterior, and long) of the AF and the frontal aslant tract (FAT) as dorsal pathways (Catani et al. 2008); and the IFOF, the ECF, and the UF as ventral pathways (Weiller et al. 2021). Although currently a lack of consensus exists about the anatomical terminations of the AF, recent anatomical models of the AF propose that this tract is divided into multiple segments differing on their cortical terminations (e.g., Catani et al. 2005; Glasser and Rilling 2008; Petrides 2014). In the present study, we performed the tractography dissection of the AF according to Catani´s model (Catani et al. 2005), one of the most used AF models and validated with post-mortem dissections (Fernández-Miranda et al. 2008). Catani et al. (2005) propose a three-segment AF model: the long segment connecting Broca´s territory with Wernicke´s territory and thus representing the classical AF; the anterior segment connecting Broca´s territory with the inferior parietal cortex; and the posterior segment linking Wernicke´s territory with the inferior parietal cortex. The three segments of the AF were dissected by creating and combining three spheric ROIs in Broca’s area, Wernicke’s area, and inferior parietal cortex following the dissection procedure described in previous articles (Catani and Thiebaut de Schotten 2012; López-Barroso et al. 2013); the ventral tracts were dissected using a two spheres approach. An anterior sphere in the extreme capsule's anterior floor common for the three ventral tracts (Catani et al. 2008; Weiller et al. 2021) to capture all the fascicles that travel between the temporal and frontal lobes through the extreme capsule. A second sphere was created to separate the fibers belonging to each of the three ventral tracts. Specifically, for the IFOF, the second sphere was located in the occipital lobe; for the UF, the second sphere was located in the temporal pole; and for the ECF, the second sphere was placed at the boundary between the occipital and temporal lobes. To separate the ECF from the IFOF, the streamlines appearing using the two spheres were further restricted with a non-ROI in the occipital lobe (and that therefore would belong to the IFOF). The dissection of the FAT was performed using two spheres as well, one in the posterior IFG-pars opercularis and another in the pre-supplementary motor area/supplementary motor area (SMA) (Catani and Thiebaut de Schotten 2012). Spurious fibers were removed from the tracks using manual ROIs. The tracks were dissected in both hemispheres. The tracks were visualized using the Surf Ice software (https://www.nitrc.org/projects/surfice). LIs were calculated for the volume of each dissected tract following the formula: (Right-Left)/(Right + Left), as performed in previous tractography studies for volume or number of streamlines (Catani et al. 2007; Thiebaut de Schotten et al. 2011; López-Barroso et al. 2013; Yazbek et al. 2021). Asymmetry was defined as left lateralization (LI < − 0.2) or right lateralization (LI > 0.2) (Springer et al. 1999; Seghier 2008).

#### Structural disconnection in P1

Since it was not possible to acquire DTI data in P1, a structural disconnection study was performed using the BCBtoolkit (Foulon et al. 2018; http://www.toolkit. bcblab.com). Specifically, the Tractotron function was used to map the normalized lesion mask of P1 onto tractography reconstructions of the white matter tracts of interest (the same tracts studied with DTI-tractrography in P2, P3, and P4) obtained from a group of healthy subjects (Rojkova et al. 2016). Tractotron measures the proportion of damage to each tract based on the overlap with the patient´s lesion mask. For further methodological details, see Thiebaut de Schotten et al. (2014) and Foulon et al. (2018).

## Results

### Lesion delineation and lesion load

**Fig. 1:**
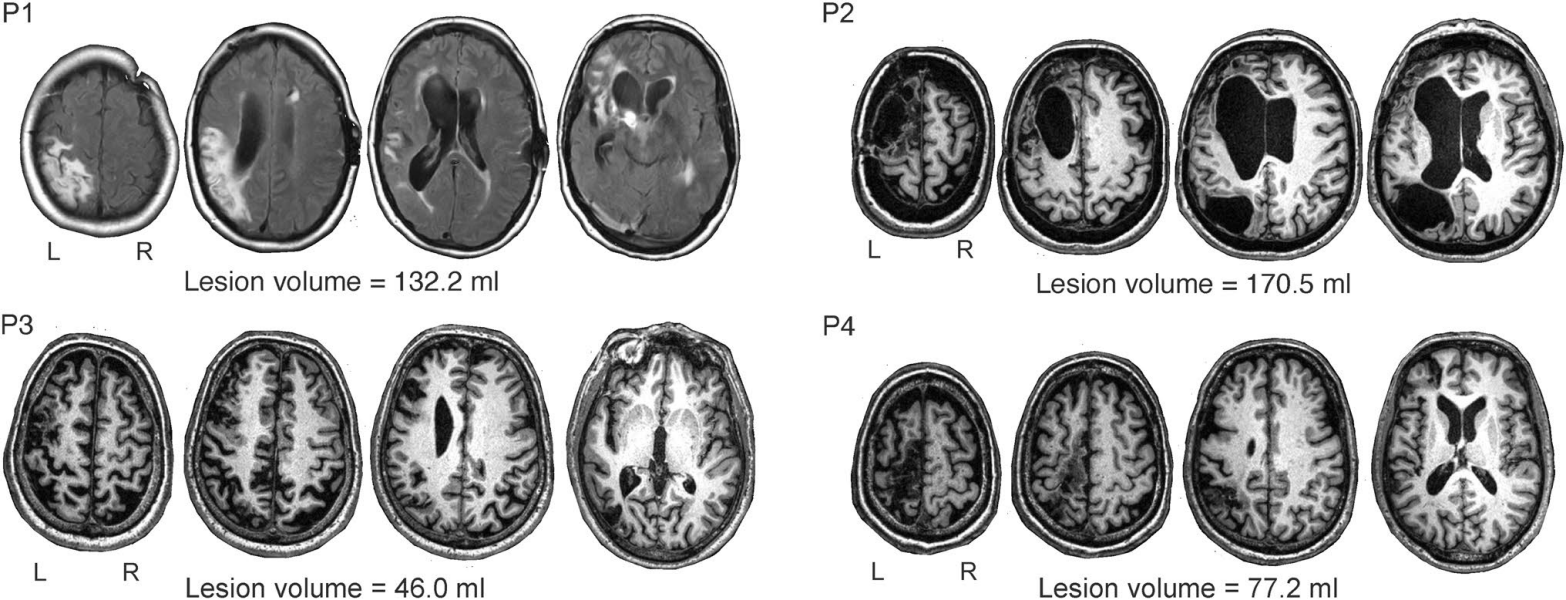
Structural MRI images showing the lesion in native space for each patient with the individual lesion volume presented below. The volume has been calculated from the image of the normalized lesion in MNI. Two white spots in P1 in the right frontal white matter substance and within the left frontal horn of the lateral ventricle correspond to the catheter for controlling hydrocephalus (further details are shown in Supplementary Results, Case Presentation). *L* left; *R* right

Structural images depicting brain damage in the left hemisphere of the four patients are shown in Fig. 1, and the percentage of damage in 21 left anatomical areas for each patient is reported in Table S3. All patients had two or more lesions. Within the perisylvian cortex, P1 had damage in the insular cortex, IFG, Rolandic operculum, sensorimotor cortex, supramarginal gyrus (SMG), superior temporal gyrus (STG), middle temporal gyrus (MTG), and angular gyrus (AG). Further damage was detected in extrasylvian anterior areas (superior frontal gyrus), but mainly affecting posterior extrasylvian areas (superior parietal cortex, superior occipital cortex, and inferior temporal cortex) as well as in the putamen with minor compromise of the thalamus. P2 presented damage in all three subregions of the IFG and inferior parietal cortex, extending into part of the STG. Extrasylvian damage affected the superior frontal gyrus and the superior parietal cortex. P3 had multiple lesions, with the largest one damaging the IFG, middle frontal gyrus (MFG), Rolandic operculum, and precentral gyrus. There were also three posterior lesions, a perisylvian one affecting the AG and two extrasylvian lesions involving the superior parietal cortex and part of the inferior temporal cortex, respectively. P4 presented perisylvian damage involving the IFG, sensorimotor cortex, and SMG. Beyond the perisylvian area, damage was found in the SMA and superior parietal cortex. Note that perisylvian damage was only partial in most of the anatomical regions studied, with less than 60% affected, except for the AG in P1 and P2 (P1:83% damaged; P2: 98% damaged), the SMG in P1 (71% damaged), and the IFG pOp in P3 (61% damaged).

### Brain activation during covert word and pseudoword repetition

**Fig. 2:**
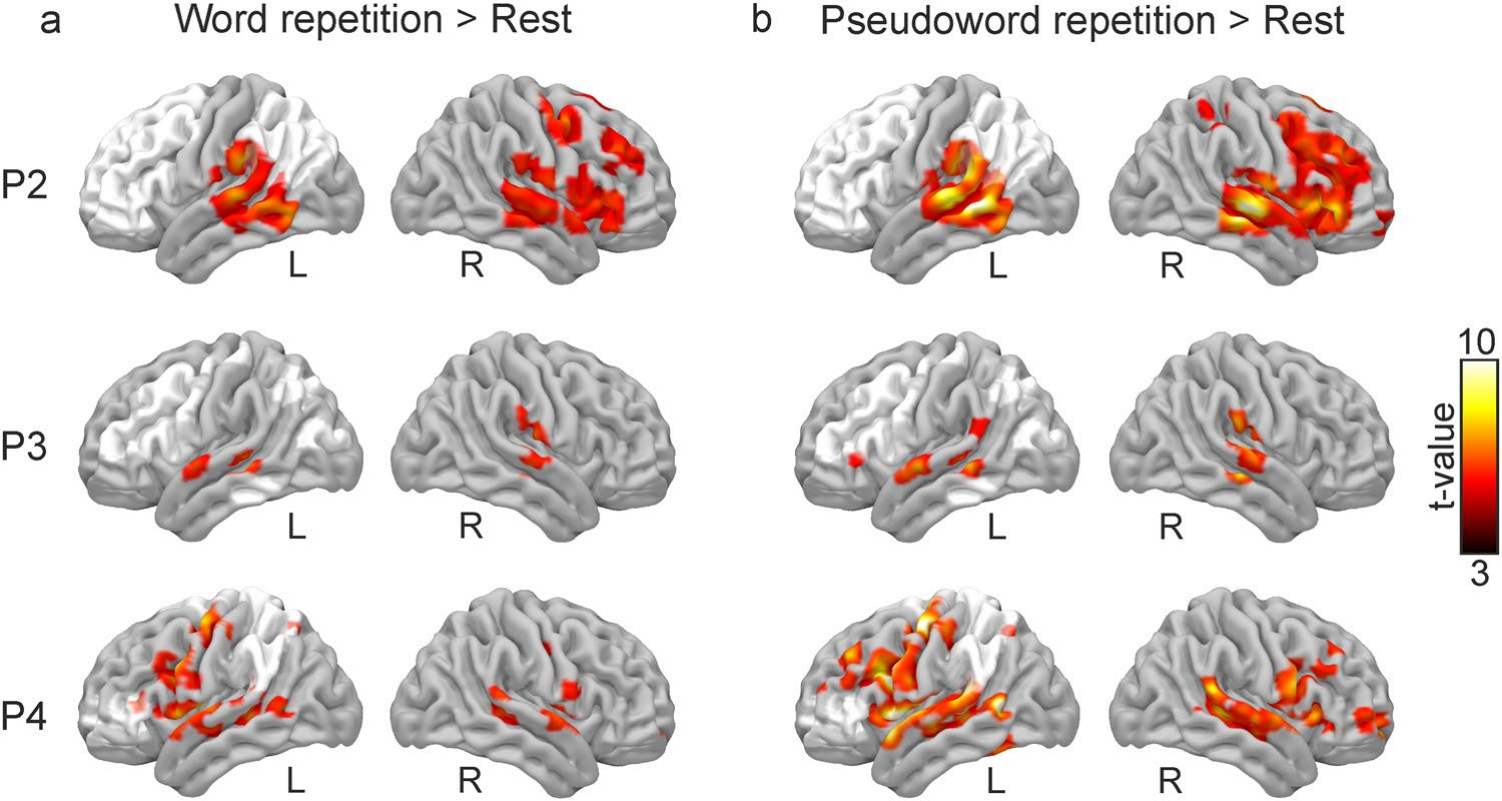
Brain activation related to word repetition (**a**); and pseudoword repetition (**b**) relative to rest in the three patients with aphasia. Lesion masks are depicted in white. *L* left; *R* right. First-level results are reported using a threshold of *p* < 0.05, FWE corrected at the cluster level

Detailed information on significant clusters, peaks, and coordinates is reported in Table S4. For P2, the comparison of the BOLD response in the word repetition > rest (Fig. 2a) and the pseudoword repetition > rest (Fig. 2b) contrasts yielded activations in a set of regions comprising the bilateral temporal areas (STG and MTG), the left SMG, and right frontal cortex areas (IFG, frontal operculum, SMA). Using hemispheric masks, LI revealed lateralization toward the right hemisphere during both word (AveLI: − 0.37) and pseudoword repetition (AveLI: − 0.36). P3 showed a more spatially restricted activation pattern. The word repetition > rest contrast revealed activations in the bilateral STG, left MTG, and right central operculum (Fig. 2a). The pseudoword repetition > rest contrast yielded bilateral activations in the STG together with activations in the left IFG, MTG, and SMG (Fig. 2b). AveLi LI suggested a symmetrical pattern of activation for word (AveLI: 0.14) and pseudoword (AveLI: 0.05) repetition. P4 showed a distributed pattern of activations. The word repetition > rest contrast revealed activations in the bilateral STG, MTG, precentral gyrus, and the left IFG, among others (Fig. 2a). The pseudoword repetition > rest contrast disclosed activations in the bilateral MTG, STG, precentral gyrus, right SMA, left MFG, and left superior parietal cortex (Fig. 2b). AveLi LI suggested a leftward activation pattern during both word (AveLI: 0.27) and pseudoword (AveLI: 0.30) repetition.

### Seed-based functional connectivity analysis

**Fig. 3:**
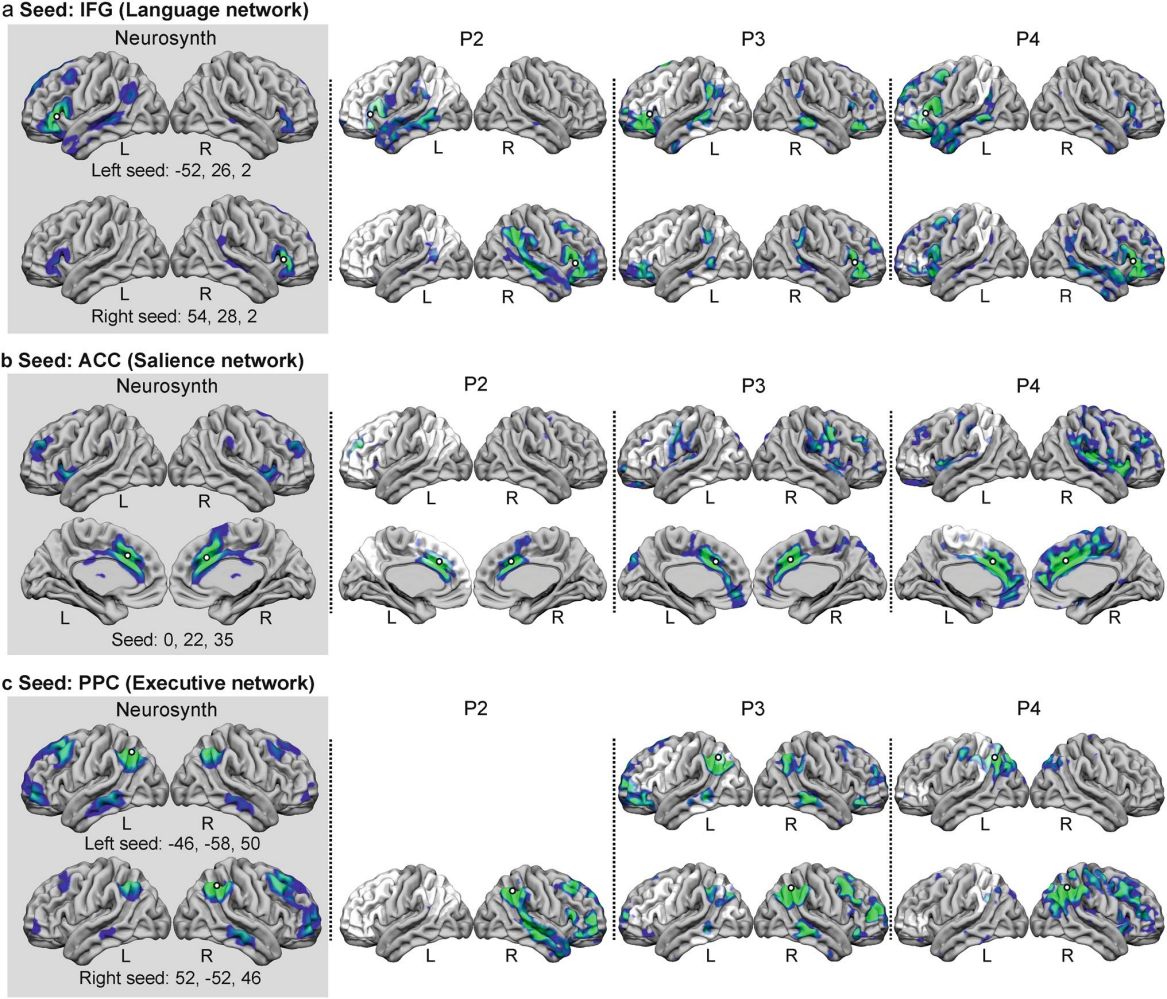
Seed-based functional connectivity results during resting state. Different resting-state networks were explored through different seeds: **a** the language network (left and right IFG), **b** the salience network (ACC), and **c** the executive network (left and right PPC). These regions were used as seed for functional connectivity analysis in the three patients and in Neurosynth neuroimaging database, which calculates the coactivation map for each seed over a sample of 1.000 healthy subjects, to obtain a measure or normal connectivity patterns. Spatial cross-correlations are reported in Table S5. Normalized lesion masks are depicted in white. A white circle with black borders indicates the location of the seed. *IFG* inferior frontal gyrus; *ACC* anterior cingulate cortex; *PPC* posterior parietal cortex; *L* left; *R* right

The language network (functional connectivity between the left IFG ROI and the rest of the brain) was found in all three studied patients (based on spatial cross-correlations with the Neurosynth results > 0.3, Table S5). All three patients mainly showed left fronto-temporal areas coactivated with the IFG (Fig. 3a), although P3 also coactivated right hemispheric regions. Neurosynth results revealed that neurotypical coactivation of this region involves a left-lateralized connectivity pattern involving the left MTG, AG, IFG, MFG, and STG, with additional involvement of the right IFG (Fig. 3a, Neurosynth, left seed). The right IFG connectivity (Fig. 3a) revealed an extended right perisylvian network mirroring the left one in all three patients (spatial cross-correlations with Neurosynth > 0.2). Visual inspection of this network in all three patients indicated greater activation in the right hemisphere than in the connectivity map obtained from Neurosynth for healthy subjects (Fig. 3a, Neurosynth, right seed), especially for P2 and P4. The salience network was also found in the three participants (spatial cross-correlations with Neurosynth > 0.26) (Fig. 3b, Table S5), although the insular cluster, which is typically observed in this network (Fig. 3b, Neurosynth), was not found in two out of three patients (P2 and P3) and it was minimal in the third one (P4). Also, the lateral cortical components involving frontal (superior frontal gyrus and IFG) and parietal areas were not present in P2, showing mainly coactivation in the midline regions surrounding the ACC seed. Finally, the executive network revealed bilateral temporo-parieto-frontal coactivation in the neurotypical brain (Fig. 3c, Neurosynth) for both seeds, left and right PPC, although coactivation was greater among ipsilateral areas. This network was retrieved in all three patients when the seed was placed in the right PPC (spatial cross-correlations with Neurosynth > 0.3, Table S5). Connectivity for the left PPC seed could not be studied in P2 because this region was completely damaged. P3 showed a network that was highly correlated with the one found in Neurosynth (spatial cross-correlations with Neurosynth = 0.42, Table S5), whereas in P4, a lower overlap with the neurotypical network was found (spatial cross-correlation with Neurosynth = 0.21, Table S5).

### ^18^FDG-PET results

**Fig. 4:**
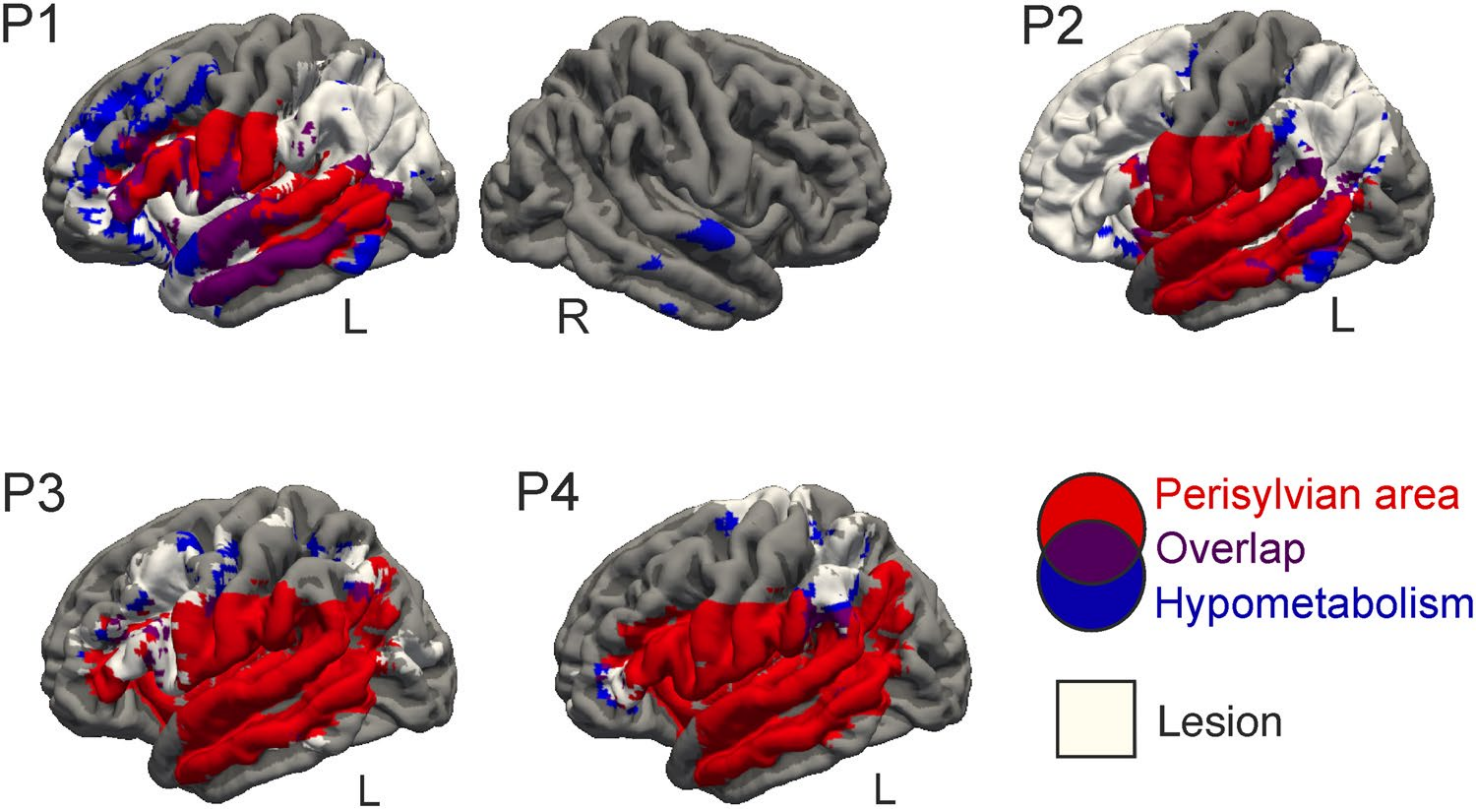
^18^FDG-PET results. ^18^FDG-PET analyses revealed areas of hypometabolism in the four patients. Compared to a group of 25 healthy controls, hypometabolic areas were found in the perisylvian (purple color) and extrasylvian areas (blue color) in perilesional regions but also in distant areas, especially in P1 and P2. A statistical threshold of *p* < 0.05 (FWE corrected) was applied. No hypermetabolic areas were found at this statistical threshold. *L* left; *R* right

^18^FDG-PET analysis revealed regions with statistically significant decreased metabolism compared to a control group in the four patients (Table S6 and Fig. 4). P1 showed significantly lower metabolic activity than controls in the left anterior insula within a cluster extending subcortically until the putamen. Reduced activity was also found in the left inferior and superior parietal cortices, and in the bilateral inferior temporal gyrus, MTG, and STG. P2 showed hypometabolic activity in the left MTG and SMG, AG, insula, and precuneus. P3 had decreased metabolic activity in the left IFG, MFG, precentral gyrus, MTG and inferior temporal gyrus. Finally, P4 showed hypometabolic activity in the SMA, SMG, IFG pTr, superior parietal cortex, and MTG. P2, P3, and P4 did not show hypometabolic activity in the right hemisphere. No hypermetabolic activity was found for any of the participants at the selected statistical threshold (i.e., *p* < 0.05 FWE corrected).

### DTI-tractography results

**Fig. 5:**
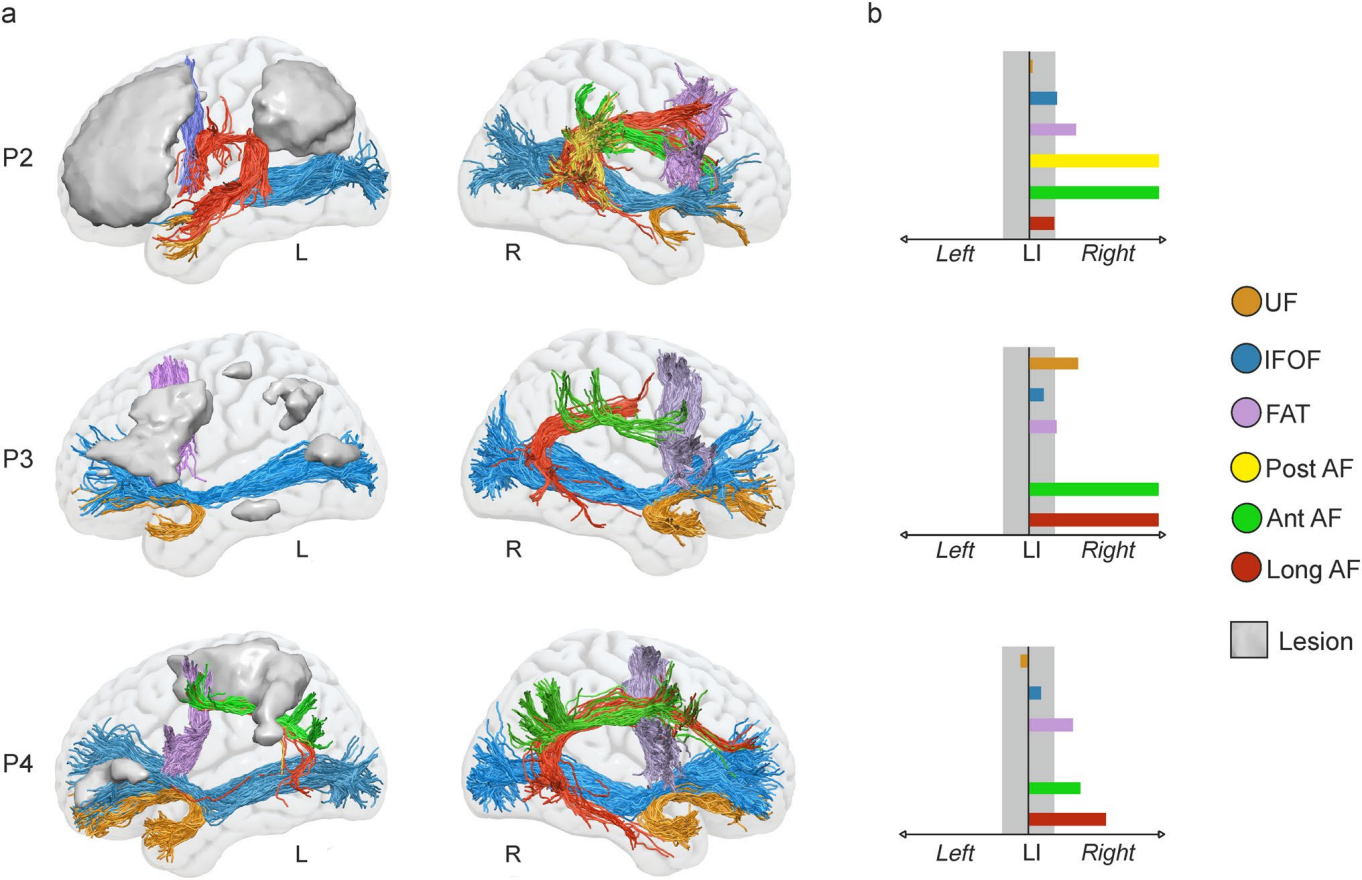
Tractography reconstruction of the dorsal and ventral white matter tracts of interest in the left and right hemisphere and lateralization indexes. **a** The tracts and the lesion mask of each patient are registered to a MNI template and rendered over a brain mesh. **b** Lateralization indexes calculated for the volume of each tract. LIs between − 0.2 and 0.2 are considered bilateral, and LIs above 0.2 are considered right lateralized. *UF* uncinate fasciculus; *IFOF* inferior frontal occipital fasciculus; *FAT* frontal aslant tract; *AF* arcuate fasciculus. *L* left; *R* right

White matter tractography performed on P2, P3, and P4 revealed different structural connectivity patterns concerning the ventral and dorsal tracts. P2 showed preserved fibers from the long AF segment in the left hemisphere, while the anterior and posterior AF segments could not be reconstructed (Fig. 5a, P2). None of the left AF segments could be reconstructed in P3 (Fig. 5a, P3). In P4, only the anterior AF segment and a few fibers of the long AF segment were reconstructed (Fig. 5a, P4). Interestingly, we also found a heterogeneous pattern in the structure of the right AF (Fig. 5a). For P2, all three AF segments were reconstructed, while in P3 and P4, only the long and anterior AF segments could be reconstructed. The FAT was reconstructed in both hemispheres in all three patients. Finally, concerning the ventral studied pathways, the UF and the IFOF were dissected in both hemispheres in all three patients. However, these tracts did not show the frontal termination in P2, being damaged at the level of the extreme capsule. The fact that its trajectory stopped at the level of the posterior floor of the extreme capsule indicates that it was the IFOF and not the inferior longitudinal fasciculus, whose fibers run more lateral to those of the IFOF and continue toward the temporal pole when those of the latter turn medially to enter the extreme capsule (Weiller et al. 2021). The ECF could not be found in any of the patients, neither in the left nor in the right hemisphere, probably because the resolution used in this study did not allow the tracking to differentiate between the streamlines belonging to the ECF and the IFOF. The LIs showed that no tract was lateralized to the left in any of the patients (Fig. 5b). All AF segments showed rightward lateralization in the three patients, except for the long AF segment that was bilateral (although trending to the right) in P2. The posterior AF segment could not be dissected in P3 and P4. The ventral tracts were bilateral in all patients, except for the UF in P3, which showed rightward lateralization. The FAT was lateralized to the right hemisphere in all patients (Fig. 5b).

### Structural disconnection results in P1

Tractotron analysis was used to explore each tract of interest's mean percentage of disconnection in P1 (Fig. S2). Regarding the left dorsal tracts (Fig. S2a), the most affected tract was the anterior segment of the AF (36.4%), followed by the posterior segment (28.8%) and the long segment (17.8%). The FAT was only affected by 6.4% (Fig. S2b). Regarding the ventral tracts studied (Fig. S2b), we found that the UF had an affectation of 34.1% and the IFOF of 19.2%.

## Discussion

MTCA is a rare aphasia profile that has garnered attention due to the unique feature of preserved repetition skills despite significant deficits in production and comprehension. In this case-series study, we investigated the function and structure of the left PSLN in four patients who presented with MTCA in the acute phase, and subsequently progressed to different aphasia subtypes with varying levels of severity in the chronic stage. Our results revealed that the lesion locations in the left PSLN and their effects on structure and function were diverse. All patients had two or more lesions affecting frontal and parietal areas, showing partial damage to the PSLN including part of the IFG, premotor cortex, inferior parietal cortex, and/or STG. The behavioral profile of MTCA persisted in P2, whereas P1, P3, and P4 evolved to less severe aphasia profiles. Below we will break down our findings.

### Clinical course of MTCA

P1 and P2 both exhibited MTCA due to large lesions in the left hemisphere (lesion volume in P1: 132.2 ml; lesion volume in P2: 170.5 ml). However, the progression of language deficits in the chronic stage resulted in different outcomes: mild aphasia in P1 (AQ-WAB-R: 82.6) and severe aphasia in P2 (AQ-WAB-R: 26). Based on WAB-R classification, in P1, the MTCA observed in the acute stage had evolved into anomic aphasia^2^; however, residual deficits of MTCA were still present such as impaired verbal working memory and sentence repetition, coupled with a moderate reduction in the frequency and quality of everyday communication (Alexander 2006; Cauquil-Michon et al. 2011). By contrast, according to the WAB-R classification in the chronic stage, P2’s language profile was still MCTA, showing impaired language performance in all domains except verbal repetition, which was virtually intact for words regardless of length, imageability, and frequency of stimuli, as well as for novel and idiomatic sentences. Explaining why P1 recovered to a mild form of aphasia while P2 remained with MCTA is a challenging task. Although lesion size is known to predict aphasia recovery (Forkel et al. 2014), both patients had large lesions. However, an age difference of 14 years at the time of stroke may explain this discrepancy, as younger patients have better prospect for recovery (Laska et al. 2001). Additionally, language impairment tends to be more pronounced on semantic tasks and WAB-R in older individuals, which may have contributed to P2’s lack of progress (Busby et al. 2022).

The two remaining patients, P3 and P4, had medium-size lesions (lesion volume in P3: 46 ml; lesion volume in P4: 77.2 ml). Consistent with previous research suggesting a positive long-term prognosis for acute post-stroke MTCA in patients with small-to-medium-size lesions (Cauquil-Michon et al. 2011; Flamand-Roze et al. 2011a, b; Jianu et al. 2021), both patients showed significant recovery of their language deficits in the chronic phase. In fact, P3 and P4 had higher AQ scores on the WAB-R than P1 and P2. These findings support the idea that preserved components of the PSLN in the language-dominant hemisphere may contribute to residual language function in post-stroke aphasia (Selnes et al. 1985; Fridriksson et al. 2010). In P3, MTCA progressed to anomic aphasia (Kertesz 2007) with features of dynamic aphasia in the chronic stage. Dynamic aphasia (Luria 1977) is characterized by preserved language functions except for propositional speech and everyday communication, which are disproportionately slowed and/or reduced compared to other language skills (Robinson et al. 1998; Barker et al. 2020). Dynamic aphasia features were elicited by applying seven specific experimental tests tapping the generation of words and connected sentences in response to verbal and non-verbal stimuli (Robinson et al. 1998) to P3 and P4. Although there are no specific criteria about the number of tests that must be abnormal to classify production deficits as belonging to dynamic aphasia, P3 was classified as having this disorder because he had marked reduced performance compared to five healthy controls in three tests (one verbal and two non-verbal). These deficits, together with impaired verbal short-term and working memory, attention, executive function, and inhibitory control may be ascribed to dysfunction associated with damage to the vicinity of the IFG and MFG (Robinson et al. 1998), and the parietal cortex (Fedorenko et al. 2012; Fedorenko and Blank 2020). In the chronic period, language deficits in P4 evolved from MTCA to latent aphasia (WAB-R AQ = 96.2) (Boller 1968), with short-term memory and working memory deficits (Silkes et al. 2021; Salis and DeDe 2022), and features of a discourse impairment (Alexander 2006). The discourse impairment was verified by impaired linguistic agility, processing speed, frequency, and quality of everyday communication and in one experimental test of story generation in response to non-verbal stimuli (Robinson et al. 1998; Alexander 2006). Findings from P3 and P4 suggest that mild affectation of the left PSLN is not enough to cause severe and lasting MTCA, but such damage can lead to less severe residuum of MTCA such as dynamic aphasia (Luria 1977; Robinson et al. 1998) and discourse impairment (Alexander 2006).

### The anatomical and functional bases of preserved verbal repetition in MTCA

One of the critical questions arising from the study of MTCA is which brain areas support the relative preservation of repetition. The isolation of speech area account proposed by Goldstein (1917) suggests that repetition in MTCA is supported by intact speech areas, including Broca´s and Wernicke´s areas and their surrounding cortex, as well as the tracts connecting them through the dorsal pathway (Geschwind et al. 1968). However, the multimodal imaging analyses performed in the current study found that both the PSLN and the dorsal pathway (i.e., the AF) were partially affected in all cases examined, both structurally and metabolically.

P1 had structural damage to the PSLN, with greater involvement of the insular cortex (53% damage) and the inferior parietal cortex (SMG [71% damage] and AG [83% damage]), and partially preserved grey matter cortical tissue in key regions for verbal repetition such as STG and IFG (IFG pOp: 11% damage; IFG: pTr 16% damage; STG: 29% damage). Yet, metabolic activity during rest, as measured with ^18^FDG-PET, was reduced in the structurally preserved mid-anterior parts of the STG and MTG, and in areas of the anterior insula extending to the IFG and ventral premotor cortex. Further, structural connectomics analysis with Tractotron revealed partial affectation of the dorsal and ventral pathways in the left hemisphere. Thus, although P1 had relatively preserved the core “language areas” that may support accurate motor articulation (Leonard et al. 2019) and feedforward/feedbackward control (Marchina et al. 2018) required for successful repetition, uncertainty remains as to whether the almost intact repetition of words, non-words, digits, and sentences in the chronic period can be sustained by a few isolated islets of healthy tissue in the left frontal and temporal areas. Therefore, an additional contribution from the homologous regions of the right hemisphere cannot be ruled out, as suggested by prior studies with H2(15)O PET activation in aphasic patients with the preserved ability for repeating words (Ohyama et al. 1996), and by the abolition of word and sentence repetition abilities after right amobarbital infusion in patients with transcortical aphasias associated to structural posterior (Bando et al. 1986) or anterior (Berthier et al. 1991) involvement of the left PSLN.

Similarly to P1, P2 had involvement of some components of the left PSLN (e.g., 98% of the AG and 55% of the IFG pTr were damaged) but preserved others, such as the anterior part of the IFG (only 18% of the IFG pOp was damaged). In addition, the direct segment of the AF—connecting canonical Broca´s and Wernicke´s areas—was found in both hemispheres, showing a symmetrical distribution in terms of volume. The anterior and posterior segments were only found in the right hemisphere. These structural findings open the possibility that repetition in this patient may be supported by the spared remnant cortex within the left PLSN and the preserved connection (long AF segment) between cortical areas in the left hemisphere. In the same line, preserved metabolism was found with ^18^FDG-PET in the left STG/MTG, the inferior sensorimotor cortex and remnants of the IFG pOp and pTr; and resting-state functional connectivity revealed that a left-lateralized fronto-temporal network was coactivated with the left IFG with significantly reduced coactivation of the AG due to its lesion. However, findings from the fMRI repetition task provided additional hints, suggesting a pivotal role for the right hemisphere in the repetition ability, in line with previous reports (Rapcsak et al. 1990; Pulvermüller and Schönle 1993; Berthier et al. 1997). Thus, although left hemispheric receptive areas in the posterior STG—which have normal metabolic activity—were recruited during word and pseudoword repetition, activity in the IFG/premotor areas was only found in the right hemisphere. In fact, activity during repetition tasks was greater in the right than the left hemisphere (i.e., right lateralized). Therefore, the left language network may not be fully functional due to the partial lesion of the IFG and inferior parietal areas adjacent to Wernicke's area. The right hemisphere's contribution may result either from greater premorbid bilateral representation of this function (Berthier 1999) or functional reorganization (Pulvermüller and Schönle 1993). In support of this argument, there is evidence that young children have a more redundant and bilateral representation of language in the brain and that with age, there is a decrease in the involvement of the right hemisphere (Olulade et al. 2020). This early redundancy makes the language system more resilient to brain damage in the left hemisphere by quickly recruiting homologues in the right hemisphere (Tuckute et al. 2022).

The other two patients, P3 and P4, had medium-size lesions (lesion volume in P3: 46 ml; lesion volume in P4: 77.2 ml). Structural and functional imaging reveals that P3 and P4 had smaller lesions and less structural involvement and/or metabolic hypofunction of components of the left PSLN (IFG, MFG or SMG) than P1 and P2. At the structural level, P3 showed frontal damage—affecting part of the IFG (pOp, pTr), MFG and precentral gyrus—and posterior lesions affecting perisylvian (i.e., 20% of the AG) and extrasylvian components (e.g., superior parietal cortex). Despite preserving critical regions of the PSLN, such as the STG, we could not dissect the left long AF in P3, which may be due to the affectation of the frontal endpoints of this tract (i.e., the premotor area). However, the left IFOF and the UF, which reach more anterior regions of the frontal cortex, were dissected in P3. Thus, the fronto-temporal interaction required to sustain verbal repetition could be achieved in P3 through the ventral pathway in the left hemisphere. Experimental studies on healthy subjects suggest that the ventral stream may support complex tasks even when they do not require semantic processing (López-Barroso et al. 2011, 2015). Healthy subjects with greater integrity of the left ventral pathway at the level of the extreme capsule are better phonological word learners during articulatory suppression conditions than those with less integrity in this pathway (López-Barroso et al. 2011), despite that auditory–motor integration processes required to learn new words relies typically on the dorsal pathway (López-Barroso et al. 2013, 2015; Assaneo et al. 2019). This flexibility in the fronto-temporal connection opens the possibility that in the presence of dorsal damage, verbal repetition may be subserved via ventral connections. This is compatible with the fact that in P3, as revealed by^18^FDG-PET, the structurally preserved PSLN also had normal metabolism at rest, whereas small clusters of hypometabolism were found in extrasylvian fronto-parietal regions. Resting-state seed-based functional connectivity also suggested that the coactivation pattern of the IFG was typical (coactivating fronto-temporo-parietal areas). Despite all this data suggesting a functional left PSLN interacting through ventral connections, the fMRI findings did not provide much evidence to support this mechanism, revealing only discrete bilateral activation during word and pseudoword repetition. Despite this, neuroimaging findings leave open the possibility that the ventral pathway could support repetition abilities in P3, possibly acting in concert with reverberating loops and short-length pathways (jumping links) in the posterior temporal cortex (Schomers et al. 2017) of one or two hemispheres.

In P4, structural damage in the PSLN comprised only part of the IFG pTr (21% of the region damaged), the postcentral (26%) and precentral gyri (15%), and the SMG (30%). These lesions also affected dorsal fronto-temporal connections. In fact, only the anterior segment of the left AF and a vestigial long AF segment could be reconstructed, whereas both ventral pathways (IFOF and the UF) were structurally preserved. ^18^FDG-PET results indicated that the spared perisylvian areas had normal metabolism at rest. Brain activation during repetition elicited a left-lateralized activation pattern, with greater left than right frontal involvement for word repetition. Resting-state functional connectivity revealed that the left IFG seed coactivated with a fronto-temporal network corresponding with the one found in Neurosynth in the healthy population. As in the case of P3, our data suggest that in P4, the repetition process may be supported by the left PSLN, relaying in the ventral pathway in the face of a damaged dorsal one. Yet, the fact that P3 and P4 had mild impairments in pseudoword repetition raised the question of whether the ventral pathway may support an optimal level of performance and under which circumstances.

In summary, our study suggests that the preserved repetition that characterizes MCTA and the types of aphasia to which each patient has progressed cannot be solely explained by the isolation of the speech area mechanism. In fact, we found variable involvement of the PSLN in all studied patients. Results suggest that repetition abilities in P1 and P2, who had large lesions that affect frontal and parietal regions, are supported by the remnant cortex and white matter tracts of the left PSLN with support of the right hemisphere, while in P2 and P3, repetition may also rely on the undamaged fronto-temporal connection and the ventral pathway.

### Multi-demand system affectation in aphasia with preserved repetition

Another relevant point is whether MTCA could be the result of a disconnection between the PSLN and extrasylvian regions belonging to the MD system. The MD cognitive system monitors executive functions, including those controlling propositional aspects of language production and auditory comprehension affected in MTCA. Resting-state functional connectivity analyses of two MD subsystems (executive and salience networks) revealed that the executive network was retrieved in the three studied patients (P2, P3, and P4; P1 could not be studied) in the right hemisphere, but only in P3 and P4 in the left hemisphere (since P2 had damaged the PPC seed region used for this analysis). P3 and P4, with a preserved executive component, showed a noticeable improvement in MTCA in the chronic period, which coincides with the good recovery reported in previous studies involving patients with MTCA and small lesions (Flamand‐Roze et al. 2011a, b). Our result also aligns well with earlier data in patients with left hemisphere stroke lesions and aphasia with poor comprehension, who achieved marked improvement in the chronic period by increasing the integration of fronto-parietal networks crucial for the cognitive control of language (Sharp et al. 2010). Spatial overlap between the left fronto-parietal network in P4 and the normative Neurosynth connectivity map revealed a lower cross-correlation index in P4 (0.21) than in P3 (0.44), and visual inspection (Fig. 3c) indicates a lack of the frontal component of this network in the left hemisphere. This could be related, for instance, to the different performance in the Hayling Sentence Completion Task (Bielak et al. 2006), showing P4 more affected performance in the initiation and suppression parts than P3. The salience network was retrieved in all three patients, but it showed a weak insular component. The FAT, a critical fascicle connecting superior and middle extrasylvian frontal areas with the IFG, did not show a significant percentage of damage in the disconnection analyses in P1. Nevertheless, the FAT was lateralized to the right hemisphere in P2, P3, and P4, suggesting a possible volume decrement in the left hemisphere. In addition, its left cortical components (SMA, IFG pOp) were functionally or structurally compromised. The FAT shrinkage in the left hemisphere would account for decreased intention to communicate and speech initiation. Moreover, reduced speech fluency during communication may have resulted from reduced synergistic interaction between FAT and the anterior segment of the AF (Marchina et al. 2011; Basilakos et al. 2014). These results all together suggest that the MD system is partially disrupted and the structural connectivity between extrasylvian and perisylvian is reduced in some cases of MTCA.

### Study limitations

This study has several limitations. First, it is important to acknowledge that this is a case-series study including only four participants with MTCA with heterogeneous lesion volumes and locations. The inclusion of this small sample resulted from the well-known low prevalence of post-stroke MTCA secondary to two or multiple left hemisphere lesions that spared the whole or most components of the PSLN. Nevertheless, since only one study used multimodal neuroimaging in a patient with thalamic hemorrhage featuring MTCA with relative preservation of brain metabolism in the left PSLN (Nitrini et al. 2019), our impetus for conducting this study in such a small sample was to further assess the structure and function of the PSLN using multimodal neuroimaging. The second limitation lies in the drawback of language evaluation in the acute-subacute periods. At that time, only clinical evaluation of language deficits was done. However, evaluations were performed during the acute-subacute period by neurologists or speech therapists, and all patients had severe verbal production and comprehension impairments with preserved repetition consistent with MTCA (Berthier et al. 1991). Formal language and cognitive evaluation at the chronic phases (7–36 months post-onset) confirmed the persistence of MTCA in one patient and the evolution to less severe aphasias in the other three. Third, regarding neuroimaging, different points should be mentioned. To start, we used a covert repetition task during fMRI, which meant we lacked control over participant´s behavioral performance inside the scanner. We chose covert repetition to minimize movement inside the scanner (Yetkin et al. 1995), which can be particularly pronounced in clinical populations. Participants were trained to silently repeat back each word or pseudoword prior to scanning to ensure task compliance. While the activation observed during covert verbal repetition can be a reliable proxy for overt repetition, it is important to acknowledge that the best way to validate a repetition task is using overt repetition, which was not feasible in our study. This limitation should be considered when interpreting our results. Regarding seed-based functional connectivity analyses during rest, results should be interpreted cautiously since: (1) participants had large-volume brain lesions, and in some cases, part of the ROIs used for analyses contained damaged tissue; and (2) the ROI used to study the language network was the IFG (as provided by CONN toolbox). Previous studies have shown that Broca's area/left IFG shows functional dissociation with a part related to domain-specific (language) functions (fronto-temporal) and another to MD functions (fronto-parietal), so having used a non-specific area may affect the results (Fedorenko et al. 2012; Bulut 2022). However, given the structural involvement of the participants, we considered appropriate to use this IFG ROI (comprising IFG pOp and IFG pTr) to ensure functional tissue in the three patients. Finally, we could not find traces of the ECF in any of the patients, probably because the current resolution has not allowed us to isolate this tract from the IFOF (whose fibers run parallel from the frontal lobe through the extreme capsule but reach more posterior regions in the occipital lobe).

## Conclusion

In the current study we assessed with multimodal neuroimaging the structural and functional neural features of four patients who developed post-stroke MTCA and progressed to different aphasia profiles. Despite the heterogeneity of the sample, our findings indicate that the preservation of verbal repetition in MTCA is not necessarily linked to the structural and functional integrity of the PSLN including its dorsal white matter connections, and that the right hemisphere and the left ventral pathway may sustain verbal repetition. The variable affectation of the PSLN, together with premorbid individual variability in language-related areas, may play a decisive role in the heterogeneous neural network recruited during repetition and the pattern of clinical evolution of MTCA. This study represents a modern conceptualization of MTCA in the light of advances in neuroscience and provides important insights into the brain bases of repetition.

## Supplementary Information

Below is the link to the electronic supplementary material.

Supplementary file1 (DOCX 5025 KB)

## Data Availability

Anonymized data will be available from the first or the corresponding author on reasonable request.

## Footnotes

1 Early labels to name mixed transcortical aphasia include *echolalia* (Pick 1898; Liepmann 1900)*, mixed echolalic aphasia* (Goldstein 1948), *isolation of the speech area* (Goldstein 1948; Geschwind et al. 1968), and *isolation syndrome* (Brown 1975; Kertesz 1979).

2 The WAB and WAB-R tend to overestimate the classification of anomic aphasia even in patients who have other type of language deficits as was the case of P1.
