# Supplementary material for: Brain structural and functional correlates of the heterogenous progression of mixed transcortical aphasia
Source: Brain Struct Funct. 2023 May 31;228(5):1347–64. doi: 10.1007/s00429-023-02655-6 (PMC10250262; doi:10.1007/s00429-023-02655-6)
Supplement: Supplementary file 1 — Supplementary file1 (DOCX 5025 KB) [file 429_2023_2655_MOESM1_ESM.docx]

*Supplementary Information*

Brain structural and functional correlates of the heterogenous progression of

mixed transcortical aphasia

*Brain Structure and Function*

Diana López-Barroso ^1,2,3,4^, José Paredes-Pacheco ^5,6^, María José Torres-Prioris ^1,2,3,4^,

Guadalupe Dávila^1,2,3,4^, Marcelo L. Berthier ^1,2,3*^

^1^Cognitive Neurology and Aphasia Unit, Centro de Investigaciones Médico-Sanitarias (CIMES), University of Malaga, Malaga, Spain

^2^Research Laboratory on the Neuroscience of Language, Faculty of Psychology and Speech Therapy, University of Malaga, Spain

^3^Instituto de Investigación Biomédica de Málaga – IBIMA, Malaga, Spain

^4^Department of Psychobiology and Methodology of Behavioural Sciences, Faculty o of Psychology, University of Malaga. Malaga, Spain

^5^Radiology and Psychiatry Department, Faculty of Medicine, Universidade de Santiago de Compostela, Spain

^6^Molecular Imaging Unit, Centro de Investigaciones Médico-Sanitarias (CIMES), General Foundation of the University of Malaga, Malaga, Spain

* Corresponding author: Marcelo L. Berthier, [mbt@uma.es](mailto:mbt@uma.es)

*Supplementary Method*

**Neuroimaging acquisition**

*Structural MRI acquisition*. For P2, P3 and P4, MRI images were obtained using a 3T MRI scanner (Philips Intera, Best, The Netherlands) equipped with an eight-channel Philips SENSE head coil. Head movements were minimized using head pads and a forehead strap. High-resolution T1 structural images of the whole brain were acquired with three-dimensional (3D) magnetization prepared rapid acquisition gradient echo (3 D MPRAGE) sequence (echo time (TE): 4.6 ms; repetition time (TR): 9.9 ms; acquisition matrix: 240/200; field of view: 240; turbo field echo (TFE) factor: 200; flip angle: 8º; reconstruction voxel size: 0.8 mm x 0.78 mm x 0.78 mm). Two hundred twenty contiguous slices with 0 mm slice gap were acquired. Axial T2 FLAIR images were acquired in a 1.5T MRI (GE Medical Systems) for P1. DWI data acquisition was performed for P2, P3 and P4, using multi-slice single-shot spin-echo planar imaging (EPI) with specific parameters as follows: FOV = 224 mm × 224 mm × 120 mm, 2 mm thick slices with no gap, reconstruction matrix = 128 voxels × 128 voxels and EPI echo train length: 59. The following parameters were used for P2: TE = 117 ms, TR = 12372 ms and 64 diffusion directions with b = 3000 s/mm2. P3 and P4 were acquired these parameters: TE = 104 ms, TR = 12500 ms, and 32 diffusion directions with b = 1500 s/mm2.

*Functional MRI acquisition.* Two different fMRI paradigms were performed: task-related fMRI and rs-fMRI*.* On the one hand, in the task-related fMRI, the brain activation pattern underlying word and pseudoword repetition was measured through two covert repetition tasks implemented in two different functional runs. Functional images were acquired with an FFE/EPI sequence with epi factor 35, TR = 3000 ms, TE = 35 ms and a flip angle of 90º. The image matrix was 64 / 64 r and the FOV was of 230 mm. 35 slices were acquired for each volume, with a 3.5 mm slice-thickness and no gap. Voxel size was 3.59 mm x 3.59 mm x 3.50 mm. *Task-related fMRI paradigm:* two functional runs, one for word repetition and another for pseudoword repetition, were used. The runs followed a block design so that each run was composed by four repetition blocks interleaved with 4 baseline-resting blocks. Each block lasted 30 seconds (10 volumes), resulting in a total of 4 minutes per run (80 volumes). In the active blocks of the word repetition run, 40 high-frequency concrete Spanish nouns were auditorily presented every 3 seconds (10 words per block). In the active blocks of the pseudoword repetition run, 40 pseudowords, derived from real words by substituting the phonemes on the basis of Spanish phonotactical rules, were auditorily presented every 3 seconds. Stimuli were presented binaurally using MRI-compatible headphones. Patients were instructed to listen carefully to the stimuli and covertly repeat words and pseudowords immediately after hearing them. They were trained outside the scanner before starting the session to ensure that they had understand the task. On the other hand, rs-fMRI involved the acquisition of 200 volumes during resting state. Patients were instructed to lie still with open eyes, to think of nothing in particular and not to fall asleep. The acquisition parameters for this FFE/EPI sequence were: TR = 3000 ms, TE = 35 ms, flip angle 82º, FOV = 220 mm, image matrix of 73 / 128 r, 50 slices with no gap. The acquisition voxel size was: 3.06 mm x 3.06 mm x 3.00 mm.

*^18^Fluorodeoxyglucose positron emission tomography (^18^FDG-PET) acquisition.* PET data acquisitions were performed for the four patients and 25 control subjects on a Discovery ST PET/CT camera (General Electric, Milwaukee, WI) after an intravenous injection of about 2 MBq/Kg in P1, 3.8 MBq/Kg in P2, 2,4 MBq/Kg in P3 and 2,5 MBq/Kg in P4. Transaxial and axial scanner resolution at the center of field of view were 6.1 and 5.6 mm full width at half maximum (FWHM), respectively. PET images were reconstructed using a 3D FORE-IR algorithm with CT attenuation correction (Matrix size, 128 x 128 x 47; voxel size, 1.95 x 1.95 x 3.27 mm).

**Neuroimaging preprocessing**

*Task-related fMRI preprocessing.* Functional MRI preprocessing followed the standard procedure implemented in the Statistical Parametric Mapping software (SPM12, <http://www.fil.ion.ucl.ac.uk/spm/>) adapted to damaged brains. The T1-image and the two fMRI runs were AC-PC oriented. Functional images of each condition (word and pseudoword repetition) were realigned to the first scan of each run. Functional scans were coregistered to the T1-images. Cost function masking was used during segmentation of the T1-image into different tissues (Brett et al. 2001). For this, the binarized lesion mask of each patient was subtracted from the T1-image, resulting in the lesioned tissue being equal to 0, and this image was the input for the segmentation process. The normalization parameters from the T1 segmentation were used for the normalization of functional images to the standard MNI space. The same parameters were used to normalize the T1-images and the lesion masks of each patient. Finally, smoothing of the functional images was carried out with an 8-mm FWHM kernel.

*Resting state fMRI functional connectivity analysis.* Resting-state functional connectivity was analyzed using the CONN functional connectivity toolbox (v.21.a, <https://web.conn-toolbox.org/>) running in MATLAB R2016b (MathWorks, Natick, MA). The preprocessing of the rs-fMRI images for each patient (P2, P3, P4) was performed following the default preprocessing pipeline of CONN toolbox (Nieto-Castanon 2020). First, all functional images (200 volumes) were AC-PC oriented using the display function of SPM. These AC-PC reoriented images were used as input to the CONN toolbox together with the structural T1-image. The T1-image that was used as input was first reoriented and cost function masking (i.e. the lesion was set to zero) (Brett et al. 2001). Briefly, the default preprocessing pipeline involved: (1) functional realignment and unwarp in which the functional volumes are coregistered and resampled to the first volume of the session. (2) Slice-timing correction to solve temporal misalignment between different slices of the functional images. (3) Outlier identification, where potential outlier scans are identified based on the amount of subject-motion during the session. (4) Direct segmentation and normalization. In this step, both the functional and the structural data are normalized to MNI space. Before normalization, segmentation of the T1-image into different tissues is performed using SPM12 unified segmentation procedure (Ashburner and Friston 2005). In the segmentation step, the lesion was included as an additional tissue class in the TPM. This was performed with the script conn_createtpm.m, resulting in a new TPM file for each patient that included a seventh tissue class characterizing the individual lesion and that will be used for segmentation and normalization, ensuring that the lesion is considered during these processes. (5) Functional smoothing using a spatial convolution with a Gaussian kernel of 8mm full width half maximum. Then, the CONN toolbox´s default denoising pipeline for rs-fMRI data, that included two steps was applied: first, an anatomical component-based noise correction procedure (aCompCor) was carried out that included noise components from white matter, CSF, the brain lesion, estimated subject-motion parameters and outliers scans or scrubbing; second, temporal frequencies below 0.01 Hz were removed from the BOLD signal to focus the analysis on slow-frequency fluctuations.

*^18^Fluorodeoxyglucose positron emission tomography (^18^FDG-PET) preprocessing:* Spatial preprocessing and statistical analysis of the PET images were performed with SPM12, running on MATLAB R2016b (Mathworks Inc., Natick, MA, United States). All T1-weighted structural images and [18F]-FDG PET images were manually aligned to anterior-posterior commissure (AC-PC) orientation. Reoriented PET images were co-registered with the T1-weighted MRI scans, except to P1 that has no T1-weighted scan. A lesion mask was drawn and applied over the T1-weighted image of P2, P3 and P4. In the case of P1, a lesion mask was drawn over a T2-weighted scan and coregistered with her PET images. Then, the PET volumes and the lesion masks were spatially normalized onto the MNI template (McGill University, Montreal, QC, Canada). The size of the resulting voxels was 2 x 2 x 2 mm. The normalized PET images were smoothed with a FWHM 8-mm Gaussian kernel. Histogram-based intensity normalization was performed using an in-house software. In this procedure, the smoothed images of each subject were divided by the mean of the normalized and smoothed images of the healthy controls. Histograms of these masked ratio images were generated, excluding damaged areas and ventricles. Finally, each smoothed PET study was divided by the most prevalent value in its ratio image.

*Diffusion tensor imaging - tractography preprocessing:* DTI data were analyzed using FSL (<http://www.fmrib.ox.ac.uk/fsl/>), MRtrix3 (<http://www.mrtrix.org/>), NiBabel (<https://github.com/nipy/nibabel>) and Trackvis software packages (<http://trackvis.org/>). The data was denoised, motion and eddy current corrected using MRtrix3 and FSL. DTI was not acquired in P1 and, thus, this analysis could not be performed in that patient. The tracts were isolated by drawing different spheres on the structural image using MRtrix3 viewer: a 10 mm radius sphere in the inferior frontal gyrus to generate the inferior fronto-occipital fasciculus (IFOF) and the extreme capsule fascicle (EFC); a 10 mm radius sphere in the superior temporal gyrus to generate the uncinate fasciculus (UF); a 15 mm radius sphere in the right inferior parietal lobule to generate the right arcuate fasciculus (AF); and a 10 mm radius sphere in the right pre-supplementary motor area/supplementary motor area (pre-SMA/SMA) to obtain the right FAT. In the case of P2, a seed ROI of the left perisylvian area after subtracting the lesioned area was obtained to generate the left AF and the left FAT. The creation of the ROI of the perisylvian area was described in the “Lesion delineation and lesion load” section in the main manuscript. In the case of P3 and P4, a 10 mm radius sphere was drawn in the left pre-SMA/SMA to obtain the left FAT; and a 15 mm radius sphere in the left inferior parietal lobule to obtain the left AF. After that, all tracks were carried out with MRtrix3 by using a set of commands which combine the Constrained Spherical Deconvolution (CSD) reconstruction method (Tournier et al. 2007) with probabilistic streamlines tractography (Tournier et al. 2010, 2012). This process significantly reduces the crossing fiber problem in diffusion images (Tournier et al. 2008). The main parameters used were: seed image: the preprocessed ROI of each case; mask: whole brain mask; tracking algorithm: iFOD2; number of generated streamlines: 50.000. NiBabel was used to transform the obtained tractograms into a readable format for Trackvis, which allowed a flexible 3D visualization of the tracts. In particular, the output of tractography generation and a b0 image were used to generate the tract-files using tck2trk tool in NiBabel.

*Supplementary Results*

**Case Presentation**

**Patient 1 (P1):** P1 was a 52-years-old right-handed woman who suffered a subarachnoid hemorrhage due to the rupture of a saccular aneurysm of the left internal carotid artery bifurcation 36 months before referral to our unit. She worked as a chief executive director in a multinational telecommunications company. The subarachnoid bleeding had a complicated course. After two unsuccessful coil embolization of the aneurysm, it was surgically clipped without immediate complications. However, two days after surgery the neurological exam disclosed that she had a severe non-fluent aphasia (hypophonic emissions restricted to monosyllables) with impaired comprehension and a right hemiparesis. Neuroimaging disclosed two large ischemic infarctions, one involving the left fronto-insular cortex extending deeply into subcortical region and the other one affecting the inferior and superior parietal lobe. She also developed hydrocephalus that was well controlled with a ventriculo-peritoneal shunt. A few days later, a bedside language evaluation revealed that P1 had a severe mixed transcortical aphasia (MTCA) characterized by a marked reduction in verbal output and impaired auditory and reading comprehension in the face of prominent echolalic repetition. She was involved in an intensive/clinic-based neurorehabilitation program. Ten months after being enrolled in neurorehabilitation, a formal language evaluation was performed using the Boston Diagnostic Aphasia Examination (BDAE) (Goodglass and Kaplan 2005). Although her language deficits had improved, the profile of language deficits was still consistent with MTCA. Spontaneous speech was non-fluent and composed of long pauses due to word retrieval difficulties. She commonly inserted filled words and stereotyped phrases (I don’t know). Syntax and grammar were preserved. Auditory comprehension was moderately impaired for word discrimination (27/37), body parts (11/20), complex material (4/12) and mildly impaired for commands (12/15). Repetition was flawlessly for words (10/10), nonwords (5/5), and sentences (10/10) and automatized sequences and melodies (days, months, counting, alphabet, singing and rhythm) were also intact. Naming was moderately impaired (Boston Naming Test: 37/60), but naming in categories was intact except for actions (7/12), animals (7/12), and tools/instruments (5/12). Language and cognitive evaluation in our unit was performed in the chronic period, 36 months post-stroke. This information is shown in Table S1.

**Patient 2 (P2):** P2 was a 66-years-old right-handed woman who suffered two simultaneous hemorrhagic lesions involving large parts of the frontal and parietal lobes. She was a highly educated woman, who previously worked as a full professor at the University. She was referred to our unit for aphasia evaluation 27 months post-onset. At the moment of the first evaluation she had severe MTCA and a residual right hemiparesis with dystonic hand posturing. Her spontaneous speech was almost nonexistent and mostly replaced by automatic echolalia and less frequently by formulaic phrases. Auditory comprehension was virtually inexistent and naming abilities was severely impaired. In contrast to her poor performance in these linguistic domains, her verbal repetition was intact and she even was able to sing without prompting preserving the lyrics and melody of several songs. Language evaluation was usually interrupted by frequent instances of task-provoked anxiety (linguistic anxiety) (Torres-Prioris et al. 2019), which was associated with recurrent phrases (“I can’t… I can’t”), a marked increase of automatic echolalia and demands to interrupt the evaluation. Further information of evaluation in the chronic period is shown in Table S1 and Torres-Prioris et al. (2019).

**Patient 3 (P3):** P3 was a 58-year-old right-handed man who in the course of 4 months suffered two strokes due to several hemorrhagic and ischemic left hemisphere infarctions presumably due to multifocal venous thrombosis. He was a manual worker. The first stroke involved the left dorsolateral prefrontal cortex and middle frontal gyrus and their underlying white matter and was associated with decreased verbal output and dysarthric repetition, but auditory comprehension and picture naming were preserved. No motor deficits were observed. The second stroke lesion involved the left temporo-parieto-occipital junction and was associated with a right homonymous hemianopia, worsening of previous verbal output deficits and new auditory comprehension deficits with preserved repetition consistent with a MTCA. The diagnosis of aphasia in the acute stage (after the second vascular episode) was established with a bedside evaluation. The patient was examined in our unit in the chronic period, seven months after the second stroke. Further information of evaluation in the chronic period is shown in Table S1.

**Patient 4 (P4):** P4 was a 58-year-old right-handed man who suffered two transient ischemic attacks with dizziness, right hemiparesis and language difficulties in two successive days. Two days later he suddenly developed right hemiparesis sparing the face with greater involvement of the leg than the arm. He was mute during two weeks and then his speech was restricted to stereotyped words (“good”, “my goodness”) and he could only understand very simple commands yet repetition was preserved, a pattern of language disorder consistent with a MTCA. On admission as inpatient in a rehabilitation center four months later, the aphasic profile of P4 was consistent with a transcortical motor aphasia with impaired comprehension. Testing with the Boston Diagnostic Aphasia Examination-Short Form (BDAE-SF) showed that his spontaneous speech was restricted to one or two words, slow, effortful, agrammatic, perseverative and containing phonemic paraphasias. Auditory comprehension was impaired (word discrimination: 34.5/37; commands: 14/15; complex ideational material: 8/12; Token Test, short-form: 19.5/36) except for auditory lexical decision (32/32) and semantic comprehension (BETA, picture-word matching: 30/30) (Cuetos Vega and González Nosti 2009). Noun naming was impaired (BDAE: 10/20, Boston Naming Test [BNT]: 46/60) and also were action naming (3/30) and both semantic fluency (4 animals in one minute) and phonemic fluency (2 words in one minute). However, automatic speech was normal and repetition was preserved for words and short-phrases. After 7 months of intensive aphasia therapy as inpatient, there was a remarkable improvement in language mostly in auditory comprehension (word discrimination: 37/37; commands: 15/15; complex ideational material: 12/12; Token Test, short-form: 35/36, and auditory lexical decision: 32/32). He was evaluated in our unit in the chronic period (12 months after onset). Further information of the evaluation in the chronic period is shown in Table S1.

**Table S1**. Demographic, clinical, language and cognitive outcomes for the four patients.

|  | **P1** | **P2** | **P3** | **P4** |
| --- | --- | --- | --- | --- |
| Age/sex | 52/F | 66/F | 58/M | 58/M |
| Handedness | Right | Right | Right | Right |
| Years of education/profession | 17/business director | 17/university professor | 10/manual worker | 17/school director |
| Etiology | Infarctions | Hemorrhages | Hemorrhagic infarctions | Infarctions |
| Number of lesions  Time of occurrence | Two  Simultaneous | Two  Simultaneous | Multiple  Successive | Two  Simultaneous |
| Acute/subacute aphasia classification* | MTCA | MTCA | 1^st^ episode: decreased verbal output  2^nd^ episode: MTCA | MTCA |
| Chronic aphasia classification**  (months after stroke onset) | Anomic aphasia  (36) | MTCA  (27) | Anomic, dynamic aphasia  (7) | Latent aphasia: discourse impairment (12) |
| ***Language and cognitive evaluation in the chronic stage*** | | | | |
| Raven Coloured Progressive Matrices/36 | 27 | 26 | 28 | 29 |
| Western Aphasia Battery-Revised |  |  |  |  |
| Fluency/10 | 8 | 0 | 8 | 9 |
| Comprehension/10 | 7.5 | 2.1 | 9.1 | 9.5 |
| Repetition/10 | 9.8 | 9.2 | 9.2 | 9.6 |
| Naming/10 | 8 | 1.7 | 9.1 | 10 |
| Aphasia Quotient/100 | 82.6 | 26 | 86.8 | 96.2 |
| Communicative Activity Log |  |  |  |  |
| Frequency/90 | 57 | 27 | 35 | 45 |
| Quality/90 | 58 | 17 | 45 | 55 |
| Total/180 | 115 | 44 | 80 | 100 |
| Word minimal pairs (words)/56 (PALPA 2) | 56 | 27 | 56 | 56 |
| Auditory lexical decision/160 (PALPA 5) | 158 | 130 | 155 | 160 |
| Spoken word-picture matching /40 (PALPA 47) | 39 | --* | 38 | 40 |
| Sentence Comprehension/60 (PALPA 55) | 42 | --* | 48 | 60 |
| Digit Span (WMS) |  |  |  |  |
| Forward | 5 | 4 | 4 | 4 |
| Backward | 2 | --* | 2 | 3 |
| Repetition: Length/24 (PALPA 7) | 24 | 24 | 24 | 24 |
| Repetition: Nonwords/24 (PALPA 8) | 24 | 21 | 12 | 11 |
| Repetition: Imag x Freq (PALPA 9) |  |  |  |  |
| Words/80 | 80 | 80 | -- | -- |
| Nonwords/80 | 76 | 55 | -- | -- |
| Cliché repetition/20 | 19 | 20 | 20 | 19 |
| Non-cliché repetition/20 | 20 | 20 | 20 | 18 |
| Phonological fluency (COWAT) | -- | --* | 9 | 18 |
| Semantic fluency (Animal fluency) | 5 | 0 | 12 | 20 |
| Trail-making test |  |  |  |  |
| Part A (sec/errors) | -- | --* | 69/2 | 44/0 |
| Part B (sec/errors) | -- | --* | > 180/4 | 67/0 |
| Hayling sentence completion task |  |  |  |  |
| Initiation (correct/latency in sec/range) | -- | -- | 15/1.17/0.62-3.54 | 13/5.98/1.38-24.14 |
| Suppression (correct/latency in sec/range) | -- | -- | 11/4.15/1.37-14.19 | 7/17.44/1.64-34.01 |
| Sentence completion (WAB-R)/10 | 10 | 2 | 10 | 10 |
| Sentence completion |  |  |  |  |
| High constraint/20 | 17 | 5 | 19 | 20 |
| Low constraint/20 | 13 | 4 | 19 | 20 |
| Echolalia (WAB-R)^δ^ | √ | √ | √ | √ |

Note: Acute aphasia classification was based on the Boston Diagnostic Aphasia Examination (BDAE) and on the BDAE Short Form (see Case Presentation in Supplementary Method). ** Chronic aphasia classification was mainly based on the Western Aphasia Battery-Revised taxonomic criteria, but diagnoses of dynamic aphasia and discourse impairment were established following Alexander criteria´s (Alexander 2006) and evaluated with specific tests devised by Robinson et al. (1998) (see Table S2). WMS-III = Wechsler Memory Scale-III. ^δ^ √ indicates instances of automatic and mitigated echolalia in auditory comprehension subtests (Yes-No Questions, Auditory Word Recognition, and Sequential Commands) of the WAB-R. COWAT indicates Controlled Oral Word Association Task. – not tested. --* tests that this patient was unable to perform due to echolalia or severe linguistic anxiety (Torres-Prioris et al. 2019a). PALPA subtests composition: Repetition: Length/24 (PALPA 7, EPLA 7). Disyllabic words with variable number of phonemes. Specifically, the test consists of 6 items of: 3 phonemes, 4 phonemes, 5 phonemes and 6 phonemes; Repetition: Nonwords/24 (PALPA 8, EPLA 7). Disyllabic pseudowords with variable number of phonemes. Specifically, the test consists of 6 items of: 3 phonemes, 4 phonemes, 5 phonemes and 6 phonemes. Repetition: Imag x Freq (PALPA 9). This test assesses the effects of imageability and frequency and their interaction in the ability to repeat. This includes the following stimuli: High-imageability/ High frequency (20 items), High-imageability/ Low frequency (20 items); Low-imageability/ High frequency (20 items); Low-imageability/ Low frequency (20 items), Non words (80 items).

**Table S2.** Experimental tests for dynamic aphasia in P3 and P4.

|  | **P3**  **Score (proportion correct)** | **P4**  **Score (proportion correct)** | **Healthy Controls**  **(n = 5)**  **Mean (SD)** | **Statistics (Crawford’s t, one tailed)**^†^ | |
| --- | --- | --- | --- | --- | --- |
|  |  |  |  | **P3** | **P4** |
| **Verbal tests** | | | | |  |
| Test A. Generation of a single word to complete a sentence  High constraint frames (max.: 20)  Low constraint frames (max.: 20) | 20 (1.0)  19 (.95) | 20 (1.0)  20 (1.0) | 19.8 ± 0.48  19.4 ± 0.89 | t=0.380; p=0.361  t= -0.410; p=0.351 | t=0.380; p=0.361  t=0.615; p=0.286 |
| Test B. Generation of a sentence from a single word (max.: 20) | 19 (.95) | 20 (1.0) | 19.4 ± 1.34 | t=-0.272; p=0.399 | t=0.615; p=0.286 |
| Test C. Generation of a sentence from a given sentence context (max.: 20) | 13 (.65) | 19 (.95) | 19.6 ± 0.89 | t=-6.77; p=0.001* | t=0.409; p=0.352 |
| **Non-verbal tests** | | | |  |  |
| Test D. Generation of a sentence from a single picture (max.: 10) | 10 (1.0) | 10 (1.0) | 10 ± 0.0 | t=0.00; p=0.5 | t=0.00; p=0.5 |
| Test E. Generation of a sentence given a pictorial scene (max.: 20) | 18 (.90) | 18 (.90) | 19 ± 1.73 | t=-0.528; p=0.313 | t=-0.528; p=0.313 |
| Test F. Generation of sentences from a pictorial scene. “what might happen next?” (max.: 20) | 4 (.20) | 20 (1.0) | 18.75 ± 0.96 | t=-14.03; p< 0.001* | t=1.18; p=0.150 |
| Test G. Story generation from a pictorial context (max.: 10) | 5 (.50) | 6 (0.60) | 9.8 ± 0.45 | t=-9.74; p< 0.001* | t=-7.71; p< 0.001* |

Note: P3 showed discourse generation deficits in response to verbal and non-verbal stimuli while P4 only showed impairments in response to non-verbal stimuli (Robinson et al. 1998) in comparison with a control sample. The control sample was composed by 5 healthy adult males individuals (age-matched, right-handed and monolingual Spanish speakers) who were part from a previous work (Berthier et al. 2021).  ^†^ Crawford et al., 2010. * Indicates p<0.05. SD: Standard deviation.

**Table S3.** Lesion load to different anatomical areas of the left hemisphere.

|  | **P1 (%)** | **P2 (%)** | **P3 (%)** | **P4 (%)** |
| --- | --- | --- | --- | --- |
| Inferior frontal gyrus pOp | 11 | 18 | 61 | 0 |
| Inferior frontal gyrus pTr | 16 | 55 | 36 | 21 |
| Inferior frontal gyrus pOb | 16 | 33 | 0 | 1 |
| Insular cortex | 53 | 0 | 0 | 0 |
| Postcentral gyrus | 17 | 2 | 3 | 26 |
| Precentral gyrus | 0 | 15 | 31 | 15 |
| Rolandic operculum | 36 | 0 | 5 | 0 |
| Supramarginal gyrus | 71 | 23 | 0 | 30 |
| Angular gyrus | 83 | 98 | 20 | 1 |
| Superior temporal gyrus | 29 | 2 | 0 | 2 |
| Middle temporal gyrus | 11 | 4 | 2 | 0 |
| Middle frontal gyrus | 6 | 67 | 17 | 3 |
| Superior frontal gyrus | 4 | 71 | 0 | 10 |
| Supplementary motor area | 0 | 42 | 0 | 60 |
| Caudate | 0 | 0 | 0 | 0 |
| Putamen | 22 | 0 | 0 | 0 |
| Thalamus | 2 | 0 | 0 | 0 |
| Superior parietal cortex | 55 | 65 | 15 | 33 |
| Precuneus | 18 | 29 | 0 | 1 |
| Superior occipital cortex | 42 | 14 | 0 | 0 |
| Inferior temporal cortex | 7 | 0 | 5 | 0 |

Note: Anatomical areas were extracted from AAL atlas using the Pickatlas toolbox (<https://www.nitrc.org/projects/wfu_pickatlas>). Lesion load is accounted as the percentage (%) of the anatomical area directly damaged by the lesion. The shadow area of the table corresponds with the anatomical areas included in the “Perisylvian area” used for PET analyses.

**Table S4**. Functional MRI activation areas for the Word repetition > Rest, and Pseudoword repetition > Rest contrasts (*p* < 0.05, FWE-corrected at the cluster level).

| **Participant** | **Contrast** | **Cluster size (n. voxels)** | **MNI coord (x, y, z)** | **Z score (peak-level)** | **Brain areas** |
| --- | --- | --- | --- | --- | --- |
| **P2** | **WR** **> Rest** | 240 | -69, -30, 26 | 7.2 | L supramarginal gyrus |
|  |  | 196 | 46, 0, 52 | 6.9 | R precentral gyrus |
|  |  | 487 | -62, -54, -4 | 6.9 | L middle temporal gyrus/L superior temporal gyrus |
|  |  | 720 | 48, 20, 0 | 6.6 | R frontal operculum/R inferior frontal gyrus pTr |
|  |  | 546 | 58, -26, 6 | 6.5 | R middle temporal gyrus/R superior temporal gyrus |
|  |  | 204 | 4, 18, 42 | 6.3 | R supplementary motor area |
|  |  | 216 | 32, 38 ,26 | 6.2 | R middle frontal gyrus |
|  | **PWR > Rest** | 1629 | -64, -40, 0 | 12.9 | L middle temporal gyrus/L superior temporal gyrus |
|  |  | 2404 | 46, 20, 0 | 12 | R frontal operculum |
|  |  | 295 | 6, 22, 64 | 7.2 | R supplementary motor area |
|  |  | 499 | 32, 36, 26 | 7 | R middle frontal gyrus |
|  |  | 384 | 52, 16, 34 | 6.93 | R middle frontal gyrus |
|  |  | 262 | -36, -64, -32 | 6.7 | L cerebellum |
|  |  | 37 | 34, -58, -32 | 5.87 | R cerebellum |
|  |  | 22 | -38, -56, -46 | 5.81 | L cerebellum |
|  |  | 23 | 6, -74, -24 | 5.6 | R cerebellum |
| **P3** | **WR > Rest** | 1235 | -61, -33, 2 | 6.5 | L superior temporal gyrus/L middle temporal gyrus |
|  |  | 1340 | 62, -18, 18 | 7.4 | R central operculum |
|  |  | 463 | -61, -3, 0 | 5.8 | L superior temporal gyrus |
|  | **PWR > Rest** | 3908 | 62, -24, 15 | 7.7 | R planum temporale/R superior temporal gyrus |
|  |  | 717 | -63, -4, -3 | 6.9 | L superior temporal gyrus |
|  |  | 1433 | -60, -36, 0 | 6.8 | L middle temporal gyrus |
|  |  | 514 | -59, -44, 20 | 6.2 | L supramarginal gyrus |
|  |  | 126 | 22, -69, -23 | 6.13 | R cerebellum |
|  |  | 51 | -57, 30, 2 | 5.3 | L inferior frontal gyrus pTr |
| **P4** | **WR > Rest** | 11430 | -56, 9, 34 | 10.9 | L precentral gyrus |
|  |  | 2268 | -42, -8, 60 | 7.8 | L precentral gyrus |
|  |  | 312 | -44, -65, -25 | 6.9 | L cerebellum |
|  |  | 118 | 31, 66, -7 | 6.9 | R frontal pole |
|  |  | 741 | 58, -6, 1 | 6.8 | R superior temporal gyrus |
|  |  | 1044 | -49, -36, 2 | 6.6 | L superior temporal gyrus |
|  |  | 301 | 68, -32, 20 | 6.5 | R superior temporal gyrus |
|  |  | 876 | -57, -58, 6 | 6.4 | L middle temporal gyrus |
|  |  | 359 | -25, -67, -20 | 6.3 | L cerebellum |
|  |  | 566 | 47, -38, 6 | 6.3 | R middle temporal gyrus |
|  |  | 472 | 54, 5, 19 | 6.3 | R precentral gyrus |
|  |  | 109 | -53, 33, 10 | 6.1 | L inferior frontal gyrus pTr |
|  |  | 213 | -27, -12, 50 | 6.1 | L precentral gyrus |
|  |  | 136 | 56, 8, -3 | 6 | R planum polare |
|  |  | 454 | 11, -73, -18 | 5.9 | R cerebellum |
|  |  | 107 | -1, -97, 0 | 5.7 | L calcarine cortex |
|  |  | 75 | 49, -9, 42 | 5.6 | R precentral gyrus |
|  |  | 54 | 32, -54, -50 | 5.6 | R cerebellum |
|  |  | 53 | -65, -27, 13 | 5.6 | L planum temporale |
|  |  | 43 | -27, -62, 54 | 5.5 | L superior parietal lobe |
|  |  | 36 | -36, 20, 5 | 5.4 | L frontal operculum |
|  | **PWR > Rest** | 26148 | -57, -56, 9 | >8 | L middle temporal gyrus |
|  |  | 3158 | -44, -10, 58 | >8 | L precentral gyrus |
|  |  | 3785 | 55, 8, 21 | >8 | R precentral gyrus |
|  |  | 812 | 30, 64, -6 | >8 | R frontal pole |
|  |  | 1869 | 28, -62, -25 | >8 | R cerebellum |
|  |  | 4315 | 39, 22, 3 | >8 | R frontal operculum |
|  |  | 667 | -43, -57, -25 | 7.74 | L cerebellum |
|  |  | 4417 | 67, -40, 20 | 7.70 | R superior temporal gyrus |
|  |  | 619 | -35, 35, 38 | 7.19 | L middle frontal gyrus |
|  |  | 252 | 4, -7, 65 | 6.75 | R supplementary motor area |
|  |  | 215 | 52, -14, -10 | 6.61 | R superior temporal gyrus |
|  |  | 107 | -31, -66, -24 | 6.54 | L cerebellum |
|  |  | 121 | -28, -11, 52 | 6.00 | L precentral gyrus |
|  |  | 103 | -45, 36, 22 | 5.99 | L middle frontal gyrus |
|  |  | 231 | 42, 51, 1 | 5.95 | R middle frontal gyrus |
|  |  | 33 | -46, 46, 8 | 5.92 | L middle frontal gyrus |
|  |  | 138 | 33, 34, 42 | 5.78 | R middle frontal gyrus |
|  |  | 41 | 47, 24, 32 | 5.71 | R middle frontal gyrus |
|  |  | 22 | -28, -61, 52 | 5.70 | L superior parietal lobe |
|  |  | 32 | -35, 56, 19 | 5.68 | L middle frontal gyrus |
|  |  | 30 | -28, 52, 18 | 5.60 | L middle frontal gyrus |
|  |  | 33 | 5, 9, 52 | 5.51 | R supplementary motor area |

Note: only the maximum peak of activation for each significant cluster is reported for each subject and contrast. Brain areas are reported based on the Neuromorphometric atlas of SPM12. WR: word repetition; PWR: pseudoword repetition.

**Table S5.** Spatial cross-correlations between each patient´s seed-based functional connectivity map and the functional connectivity map derived from Neurosynth database in standard space.

| Neurosynth FC maps |  | P2 P3 P4 | | | | | | | | | | | | | | | |
| --- | --- | --- | --- | --- | --- | --- | --- | --- | --- | --- | --- | --- | --- | --- | --- | --- | --- |
| ROI |  | | ROI | | | | |  |  |  |  |  |  |  |  |  |  |
|  | **1** | **2** | | **3** | **4** | **5** | **1** | | **2** | **3** | **4** | **5** | **1** | **2** | **3** | **4** | **5** |
| **1** | .33 |  | |  |  |  | .36 | | .28 |  | .27 |  | .43 | .26 |  |  |  |
| **2** |  | .32 | |  |  | .24 | .25 | | .36 |  |  |  | .22 | .28 |  |  |  |
| **3** |  |  | | .37 |  |  |  | |  | .27 |  |  |  |  | .37 |  |  |
| **4** |  |  | |  |  |  | .23 | |  |  | .42 | .37 |  |  |  | .21 | .21 |
| **5** |  | .23 | |  |  | .34 |  | |  |  | .33 | .44 |  |  |  |  | .31 |

Note: only correlations above equal or above 0.2 are reported in this table. FC: functional connectivity; ROI 1: left inferior frontal gyrus; ROI 2: right inferior frontal gyrus; ROI 3: anterior cingulate cortex; ROI 4: left posterior parietal cortex; ROI 5: right posterior parietal cortex.

**Table S6.** ^18^FDG-PET analyses results corrected for multiple comparisons (*p* < 0.05, FWE-corrected at the cluster level).

| Participant | Contrast | Cluster size (n. voxels) | MNI coord  (x, y, z) | Z score (peak-level) | Brain areas |
| --- | --- | --- | --- | --- | --- |
| P1 | Hypometabolism | 9748 | -30, 12, 2 | < 8 | L anterior insular cortex |
|  |  | 206 | -10, -26, 38 | 6.13 | L cingulate gyrus |
|  |  | 15 | -44, -34, 50 | 6.09 | L supramarginal |
|  |  | 16 | -34, -40, 56 | 5.99 | L superior parietal cortex |
|  |  | 21 | -24, -56, 60 | 5.76 | L superior parietal cortex |
|  |  | 12 | -62, -30, 20 | 5.45 | L supramarginal |
|  |  | 131 | 62,-4, -4 | 5.41 | R inferior temporal gyrus |
|  |  | 110 | 60, -20, -28 | 5.37 | R middle temporal gyrus |
|  |  | 24 | -14, 54,-8 | 5.19 | L medial frontal gyrus |
|  |  | 21 | 54, 0, -30 | 5.17 | R middle temporal gyrus |
|  |  | 24 | 40, -56, -48 | 5.16 | R cerebellum |
|  |  | 10 | -38, -24, 12 | 5.04 | L transverse temporal gyrus |
| P2 | Hypometabolism | 5420 | -32, 12, 0 | < 8 | L anterior insular cortex |
|  |  | 932 | -44, -66, 22 | 7.14 | L angular gyrus |
|  |  | 72 | -24, -74, 34 | 6.62 | L superior parietal cortex |
|  |  | 16 | -10, -52, 56 | 5.74 | L precuneus |
|  |  | 35 | 38, -60, -48 | 5.28 | R cerebellum |
|  |  | 22 | -58, -12, -22 | 5.02 | L middle temporal gyrus |
|  |  | 16 | -62, -30, -10 | 4.96 | L middle temporal gyrus |
| P3 | Hypermetabolism | 749 | -46, -4, 44 | 7.49 | L precentral gyrus |
|  |  | 218 | -50, 14, 20 | 6.40 | L inferior frontal gyrus pOp |
|  |  | 35 | -44, 28, 10 | 5.98 | L inferior frontal gyrus pTr |
|  |  | 21 | -40, 32, 20 | 5.69 | L middle frontal gyrus |
|  |  | 10 | -52, 6, 18 | 5.36 | L precentral gyrus |
|  |  | 13 | -62, -38, -14 | 5.19 | L middle temporal gyrus |
| P4 | Hypermetabolism | 711 | -6, -6, 60 | 7.46 | L supplementary motor cortex |
|  |  | 757 | -56, -42, 36 | 7.19 | L supramarginal gyrus |
|  |  | 191 | -44, 36, 0 | 6.48 | L inferior frontal gyrus pTr |
|  |  | 10 | -22, -54, 60 | 5.68 | L superior parietal cortex |
|  |  | 19 | -58, -42, 2 | 5.39 | L middle temporal gyrus |

Note: Hypometabolism results from the contrast Patient < Controls.

**Figure S1**

**
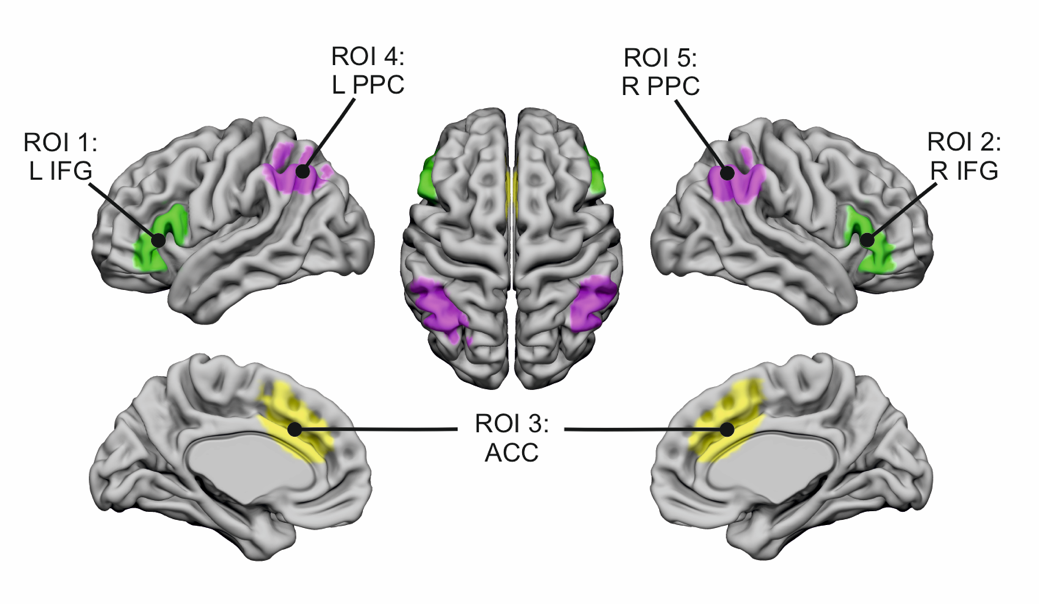
**

**Fig. S1.** Regions of interest (ROIs) used to explore the networks of interest. ROIs were selected from CONN toolbox. L: left; R: right; IFG: inferior frontal gyrus; ACC: anterior cingulate cortex; PPC: posterior parietal cortex.

**Figure S2**


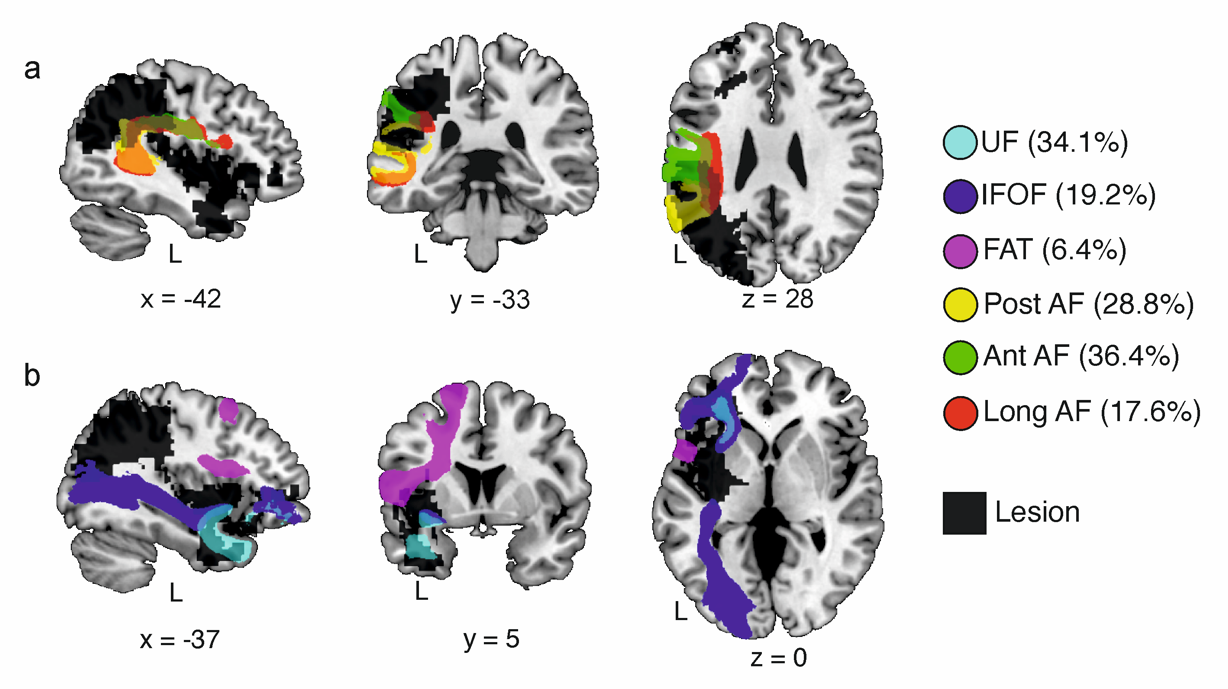


**Fig. S2** Lesion based disconnection analysis in patient 1 (P1). (a) Overlap between the normalized lesion mask of P1 (black) and the probabilistic templates of the three segments of the arcuate fasciculus. (b) Overlap between the normalized lesion mask of P1 (black) and the templates of the FAT, the IFOF, and the UF. Probabilistic templates of the white matter tracts were extracted from Tractotron white matter atlas and plotted using a threshold of 70%. The percentage of damage for each white matter tract is shown, as calculated with Tractotron function implemented in BCB toolkit (http://www.toolkit. bcblab.com)

*Supplementary References*

Alexander MP (2006) Impairments of procedures for implementing complex language are due to disruption of frontal attention processes. J Int Neuropsychol Soc 12:236–247. https://doi.org/10.1017/S1355617706060309

Ashburner J, Friston KJ (2005) Unified segmentation. Neuroimage 26:839–851

Brett M, Leff AP, Rorden C, Ashburner J (2001) Spatial Normalization of Brain Images with Focal Lesions Using Cost Function Masking. Neuroimage 14:486–500. https://doi.org/10.1006/NIMG.2001.0845

Crawford JR, Garthwaite PH, Porter S (2010) Point and interval estimates of effect sizes for the case-controls design in neuropsychology: Rationale, methods, implementations, and proposed reporting standards. Cogn Neuropsychol 27:245–260. https://doi.org/10.1080/02643294.2010.513967

Cuetos Vega F, González Nosti M (2009) BETA : Batería para la Evaluación de los Trastornos Afásicos : manual

Goodglass H, Kaplan E (2005) Boston Diagnostic Aphasia Examination- Third Edition- spanish version. Editorial Médica Panamericana

Nieto-Castanon A (2020) FMRI minimal preprocessing pipeline. In: Handbook of functional connectivity Magnetic Resonance Imaging methods in CONN. Hilbert Press, pp 3–16

Robinson G, Blair J, Cipolotti L (1998) Dynamic aphasia: an inability to select between competing verbal responses? Brain a J Neurol 121:77–89

Torres-Prioris MJ, López-Barroso D, Paredes-Pacheco J, et al (2019a) Language as a threat: Multimodal evaluation and interventions for overwhelming linguistic anxiety in severe aphasia. Front Psychol 10:. https://doi.org/10.3389/fpsyg.2019.00678

Tournier J-D, Calamante F, Connelly A (2007) Robust determination of the fibre orientation distribution in diffusion MRI: non-negativity constrained super-resolved spherical deconvolution. Neuroimage 35:1459–1472

Tournier J-D, Yeh C-H, Calamante F, et al (2008) Resolving crossing fibres using constrained spherical deconvolution: validation using diffusion-weighted imaging phantom data. Neuroimage 42:617–625

Tournier J, Calamante F, Connelly A (2012) MRtrix: diffusion tractography in crossing fiber regions. Int J Imaging Syst Technol 22:53–66

Tournier JD, Calamante F, Connelly A (2010) Improved probabilistic streamlines tractography by 2nd order integration over fibre orientation distributions. In: Proceedings of the international society for magnetic resonance in medicine. John Wiley & Sons, Inc. New Jersey, USA
